# Concurrent inhibition of p300/CBP and FLT3 enhances cytotoxicity and overcomes resistance in acute myeloid leukemia

**DOI:** 10.1038/s41401-025-01479-w

**Published:** 2025-01-30

**Authors:** Yu-jun Chen, Yu Zhao, Ming-yue Yao, Ya-fang Wang, Ming Ma, Cheng-cheng Yu, Hua-liang Jiang, Wu Wei, Jie Shen, Xiao-wei Xu, Cheng-ying Xie

**Affiliations:** 1 School of Life Science and Technology, ShanghaiTech University, Shanghai, 201210 China; 2 Shanghai Institute for Advanced Immunochemical Studies, ShanghaiTech University, Shanghai, 201210 China; 3 Lingang Laboratory, Shanghai, 200031 China; 4 School of Life Sciences and Biotechnology, Shanghai Jiao Tong University, Shanghai, 200240 China; 5 Drug Discovery and Development Center, Shanghai Institute of Materia Medica, Chinese Academy of Sciences, Shanghai, 201203 China; 6 Department of Pharmacy, The SATCM Third Grade Laboratory of Traditional Chinese Medicine Preparations, Shuguang Hospital Affiliated to Shanghai University of Traditional Chinese Medicine, Shanghai, 201203 China; 7 Department of Hematology, Shanghai Jiao Tong University School of Medicine Affiliated Shanghai General Hospital, Shanghai, 200080 China

**Keywords:** acute myeloid leukemia, FLT3, p300/CBP, quizartinib resistance, combination strategy

## Abstract

FMS-like tyrosine kinase-3 (*FLT3*), a class 3 receptor tyrosine kinase, can be activated by mutations of internal tandem duplication (FLT3-ITD) or point mutations in the tyrosine kinase domain (FLT3-TKD), leading to constitutive activation of downstream signaling cascades, including the JAK/STAT5, PI3K/AKT/mTOR and RAS/MAPK pathways, which promote the progression of leukemic cells. Despite the initial promise of FLT3 inhibitors, the discouraging outcomes in the treatment of FLT3-ITD-positive acute myeloid leukemia (AML) promote the pursuit of more potent and enduring therapeutic approaches. The histone acetyltransferase complex comprising the E1A binding protein P300 and its paralog CREB-binding protein (p300/CBP) is a promising therapeutic target, but the development of effective p300/CBP inhibitors faces challenges due to inherent resistance and low efficacy, often exacerbated by the absence of reliable clinical biomarkers for patient stratification. In this study we investigated the role of p300/CBP in FLT3-ITD AML and evaluated the therapeutic potential of targeting p300/CBP alone or in combination with FLT3 inhibitors. We showed that high expression of p300 was significantly associated with poor prognosis in AML patients and positively correlated with FLT3 expression. We unveiled that the p300/CBP inhibitors A485 or CCS1477 dose-dependently downregulated FLT3 transcription via abrogation of histone acetylation in FLT3-ITD AML cells; in contrast, the FLT3 inhibitor quizartinib reduced the level of H3K27Ac. Concurrent inhibition of p300/CBP and FLT3 enhanced the suppression of FLT3 signaling and H3K27 acetylation, concomitantly reducing the phosphorylation of STAT5, AKT, ERK and the expression of c-Myc, thereby leading to synergistic antileukemic effects both in vitro and in vivo. Moreover, we found that p300/CBP-associated transcripts were highly expressed in quizartinib-resistant AML cells with FLT3-TKD mutation. Targeting p300/CBP with A485 or CCS1477 retained the efficacy of quizartinib, suggesting marked synergy when combined with p300/CBP inhibitors in quizartinib-resistant AML models, as well as primary FLT3-ITD^+^ AML samples. These results demonstrate a potential therapeutic strategy of combining p300/CBP and FLT3 inhibitors to treat FLT3-ITD and FLT3-TKD AML.

## Introduction

Acute myeloid leukemia (AML) is a highly aggressive hematological malignancy characterized by impaired differentiation and clonal proliferation of myeloid precursor cells [1, 2]. Genomic investigations have identified recurrent genetic alterations in AML, including amplifications, deletions, rearrangements, and point mutations [1]. This disease predominantly affects patients over 60 years old and has a generally poor prognosis [3, 4]. In addition, the standard therapies for AML have remained unchanged for the last 20 years, highlighting the urgent need for novel therapeutics to improve the long-term outlook of this challenging disease. FMS-like tyrosine kinase-3 (*FLT3*), a class 3 receptor tyrosine kinase, can be activated by mutations of internal tandem duplication (FLT3-ITD) or point mutations in the tyrosine kinase domain (FLT3-TKD), leading to constitutive activation of downstream signaling cascades, including the JAK/STAT5, PI3K/AKT/mTOR and RAS/MAPK pathways, which promote the progression of leukemic cells [5, 6]. Over 30% of AML patients carry FLT3 mutations, with ITD mutations occurring in the juxta-membrane domain in up to 25% of patients, which contributes to the risk of disease relapse and poor overall survival (OS) [7–9]. FLT3 inhibitors targeting FLT3-ITD mutations, FLT3-TKD mutations, or both, have demonstrated efficacy as monotherapies in clinical trials [10, 11]. Notably, second-generation FLT3 inhibitors, such as quizartinib, a potent type II inhibitor that selectively targets the FLT3-ITD mutation [12], and gilteritinib, a type I inhibitor that targets FLT3-ITD and TKD mutations [13], have been demonstrated to improve OS and increase remission rates in patients with relapsed or refractory (R/R) FLT3-mutated AML, compared with conventional chemotherapy [14–18]. With excellent potency, selectivity, and pharmacokinetic properties, quizartinib is the preferred frontline drug candidate for patients with newly diagnosed FLT3-ITD AML [19, 20]. However, the clinical responses are often limited and short-lived due to acquired resistance after prolonged exposure, leading to disease relapse [21–25]. Primary and acquired resistance to FLT3 inhibitors still presents a formidable challenge, underscoring the imperative of integrating FLT3 inhibitors with other therapeutic modalities, both conventional and investigational, to address this issue effectively [26].

AML is a fatal hematological malignancy characterized by abnormal transcriptional reprogramming and driven by a multitude of heterogeneous mutations, with the mutation of epigenetic regulators being a central and recurrent theme. The E1A binding protein P300 (*EP300*, also known as p300) and its paralog CREB-binding protein (*CREBBP*, also known as CBP) are highly conserved histone acetyltransferases (HATs) that participate in many physiological and pathological processes [27, 28]. p300/CBP plays an essential role in maintaining hematopoiesis through interactions with key transcription factors like GATA1, MYB and AML-1 [29–31]. Rearrangements of p300 or CBP with the MOZ gene in AML, namely MOZ-p300 and MOZ-CBP, are associated with the generation of leukemogenic fusion proteins and tumorigenesis [32]. To date, significant progress has been made in the development of p300/CBP inhibitors, with a focus on targeting the bromodomain and HAT domains [33, 34]. Notably, compounds like A485 [35] and inobrodib (CCS1477) [32, 36] have demonstrated promising anticancer activities against hematological malignancies; however, none of these inhibitors have received FDA approval yet. The primary obstacle lies in drug resistance and the limited efficacy of eradicating leukemic stem cells, necessitating synergistic therapeutic approaches. Additionally, suppression of p300/CBP-mediated global histone acetylation does not consistently induce an antiproliferative phenotype [35]. Therefore, investigating potential biomarkers of sensitivity to p300/CBP inhibitors in clinical settings and enhancing patient selection for this targeted therapy are crucial.

In the present study, we investigated the role of p300/CBP in FLT3-ITD AML and aimed to evaluate the therapeutic potential of targeting p300/CBP alone or in combination with FLT3 inhibitors. Our findings revealed that high expression of p300 was associated with poor survival in AML patients and positively correlated with FLT3. Treatment with p300/CBP inhibitors demonstrated notable efficacy against FLT3-ITD AML and effectively overcame resistance to the FLT3 inhibitor quizartinib. When combined with quizartinib, p300/CBP inhibitors exhibited synergistic effects in both FLT3-ITD AML xenograft models and AML patient samples by simultaneously suppressing H3K27 acetylation, c-Myc and FLT3 pathways. On the basis of these results, we hypothesized that coinhibition of p300/CBP and FLT3 may have therapeutic value in FLT3-ITD AML through targeting both epigenetic mechanisms and receptor tyrosine kinase signaling.

## Materials and methods

### Reagents and antibodies

Quizartinib (#HY-13001) was purchased from MedChemExpress (New Jersey, USA). A485 (#1889279-16-6) and CCS1477 (#2222941-37-7) were purchased from Bidepharm (Shanghai, China). Primary antibodies against the following proteins were purchased from Cell Signaling Technology (Danvers, MA, USA): FLT3 (#3462), P-FLT3 (Tyr589/591, #60413), STAT5 (#25656), P-STAT5 (Tyr694, #4322), AKT (#4685), P-AKT (Ser473, #4060), ERK1/2 (#4695), P-ERK1/2 (Thr202/Tyr204, #9101), c-Myc (#18583), Cyclin D1 (#55506), CDK2 (#18048), P-CDK2 (Thr160, #2561), p27 (#3686), p300 (#86377), acetyl-histone H3 (Lys27, #8173), histone H3 (#4499), β-Tubulin (9F3, #2128), GAPDH (D16H11, #5174) and β-Actin (13E5, #4970). The goat anti-mouse IgG (#3153977) and goat anti-rabbit IgG (#3135933) secondary antibodies were purchased from Merck/Millipore (Billerica, MA, USA).

### Cell lines and cell culture

The MV-4-11 and RS4;11 cell lines were purchased from the American Type Culture Collection (ATCC, Manassas, VA, USA). The MOLM-13, EOL-1, HEL and SET-2 cell lines were purchased from DSMZ (Braunschweig, Germany). The quizartinib-resistant cell line MV-4-11/quizartinib was constructed by continuous incubation with increasing concentrations of quizartinib for several months. All the cell lines were cultured in RPMI-1640 medium (Gibco, New York, USA) supplemented with 10% fetal bovine serum (FBS, Gibco) and 1% penicillin/streptomycin (MeilunBio, Dalian, China) and maintained at 37 °C in a 5% CO_2_ incubator. The cell lines were authenticated by Geneing Biotechnologies Inc. (Shanghai, China).

### Cell proliferation assay

The experiments were carried out in triplicate in 96-well plates. After the cells were incubated with gradient dilutions of drugs for 3 or 5 days, cell viability was detected using the MTT assay (Sigma-Aldrich, St. Louis, MO) or Cell Counting Kit-8 (CCK8, MeilunBio) [37]. The half-maximal inhibitory concentration (IC_50_) values were calculated using Prism 5 curve-fitting software (GraphPad Software, San Diego, CA). The combination index (CI) was calculated using CalcuSyn software (Calcusyn, Inc., Paramus, NJ), which defines CI < 1 as synergistic, CI = 1 as additive, and CI > 1 as antagonistic.

### Cell cycle analysis

The cells were plated in 12-well plates at a density of 1 × 10^6^ cells/well and treated with the specified concentrations of drugs for 24 h. After washing with PBS, the cells were permeabilized with 70% cold ethanol overnight, followed by treatment with RNase A and propidium iodide (Vazyme Biotech Co., Ltd., Nanjing, China) for 30 min at room temperature. The cell cycle distribution was analyzed using a FACSCalibur flow cytometer (BD Pharmingen, San Diego, CA, USA).

### Western blotting

After treatment, the cells were harvested with SDS lysis buffer and subjected to 6%–15% SDS-PAGE. The proteins were transferred onto polyvinylidene difluoride membranes (Millipore, Bedford, MA). After blocking with 5% nonfat milk, the membranes were incubated with primary antibodies at 4 °C overnight, followed by incubation with secondary antibodies at room temperature for 2 h. Target protein bands were detected by the enhanced chemiluminescence and imaged with Tanon 4600 imaging system (Tanon, Shanghai, China).

### Survival and correlation analyses

Survival analyses of AML patients stratified by *EP300* expression, and correlation analyses of *EP300* and *FLT3* in the TCGA-acute myeloid leukemia-like (LAML) cohort were performed via the Gene Expression Profiling Interactive Analysis (GEPIA) web service (http://gepia.cancer-pku.cn/index.html).

### RNA extraction and quantitative real-time PCR (RT-qPCR)

Total RNA from cells was extracted using RNAiso Plus reagent (Takara, Tokyo, Japan), followed by reverse transcription using HiScript II qRTSuperMix (Takara) according to the manufacturer’s protocol. RT-qPCR was performed using AceQ qPCR SYBR Green Master Mix (Takara), and gene expression was detected using quantitative RT-PCR in a Quant Studio 7 Flex Real-Time PCR system (ABI, Waltham, USA). The expression levels of the target genes were normalized to those of GAPDH and calculated using the ∆∆Ct method. The detailed primer sequences are listed in Table S1.

### Chromatin immunoprecipitation (ChIP)-qPCR assay

The chromatin immunoprecipitation (ChIP) assay was performed using a Simple ChIP Plus Enzymatic Chromatin IP Kit (#9005, Cell Signaling Technology) according to the manufacturer’s instructions. The cells were crosslinked with formaldehyde and lysed, and the harvested chromatin was fragmented by sonication. The fragmented chromatin was subsequently incubated overnight with antibodies against acetyl-histone H3 (Lys27) or normal rabbit IgG as a control. Immunoprecipitated DNA was analyzed by RT-qPCR to detect the enrichment of H3K27Ac at the promoter region of *FLT3*. The detailed primer sequences used for ChIP-qPCR are listed in Table S2.

### Small interfering RNA (siRNA) transfection

siRNAs targeting p300 or c-Myc were purchased from Tsingke (Beijing, China) and then transfected into MV-4-11 and MOLM-13 cells with Lipofectamine RNAIMAX (Invitrogen, Thermo Fisher Scientific, USA). The sequence of sip300 is 5′-GGACUACCCUAUCAAGUAATT-3′. The sequences of sic-Myc are #1 5′-GUGCAGCCGUAUUUCUACUTT-3′ and #2 5′-GAACACACAACGUCUUGGATT-3′.

### Coimmunoprecipitation (Co-IP)

For the Co-IP assay, after treatment, the cells were lysed in NP40 buffer (50 mM Tris-HCl, pH 7.4, 150 mM NaCl_2_, 1% Nonidet P40, 5 mM EDTA, and complete protease inhibitor mix, Roche) on ice for 30 min. After centrifugation and quantification, equal amounts of total proteins were immunoprecipitated with the indicated antibodies (Santa Cruz, p300, sc-32244; control IgG antibody, sc-52336) overnight at 4 °C, followed by the addition of 30 μL protein A/G agarose beads (Thermo Scientific, 20421; only for IP with unconjugated antibodies) for 4 h. After washing with NP40 buffer, the precipitates were eluted by boiling with 1×SDS lysis buffer and analyzed by Western blotting. An aliquot of each lysate was used as an input control.

### RNA sequencing (RNA-seq) analysis

After treatment with DMSO or 20 nM quizartinib for 6 h, MV-4-11 cells or MV-4-11/quizartinib cells were collected for RNA sequencing. Total RNA was extracted using RNAiso Plus (Takara) and RNA sequencing was performed by Shanghai Majorbio Biopharm Biotechnology Co., Ltd. (Shanghai, China). To identify differentially expressed genes (DEGs) between two different samples, the expression level of each transcript was calculated according to the transcripts per million reads (TPM) method. RSEM was used to quantify gene abundances. Differential expression analysis was performed using DESeq2. DEGs with a |log2FC | ≥ 1 and an FDR ≤ 0.05 were considered target genes. In addition, GO and KEGG functional enrichment analyses were performed by GOATOOLS and KOBAS, respectively.

### Mouse xenograft models

Animal experiments were conducted in accordance with the guidelines of the Institutional Animal Care and Use Committee of the Shanghai Institute of Materia Medica, Chinese Academy of Sciences and Lingang Laboratory (Shanghai, China). Five- to six-week-old female athymic BALB/c nude mice were purchased from Charles River Laboratories (Beijing, China). A total of 1 × 10^7^ cells suspended in 100 μL of saline solution were inoculated subcutaneously into the right flank. The mice bearing established xenografts were randomly assigned to four groups when the tumor volumes reached ~100 mm^3^. Each group (*n* = 5) was treated daily with vehicle, 1 mg/kg quizartinib (oral gavage), 100 mg/kg A485 (intraperitoneally), or a combination of both agents. Body weights and tumor sizes were measured every two days, and tumor volumes were calculated as (length × width^2^)/2. After the mice were euthanized at the end of the experiment, the tumor weights were measured, and the tumor tissues were harvested and processed for Western blot analysis.

### Primary AML patient samples

Each participant provided written informed consent, and the study was approved by the Ethics Committee of Shanghai Jiao Tong University School of Medicine Affiliated Shanghai General Hospital, following the principles of the Declaration of Helsinki of 1975. Bone marrow mononuclear cells (BMMCs) or peripheral blood mononuclear cells (PBMCs) were isolated using Ficoll-Hypaque density gradient centrifugation (Cedarlane Laboratories, Canada). Red blood cells were lysed with ACK lysing buffer (Invitrogen). BMMCs and PBMCs were cultured in RPMI-1640 medium and Iscove’s Modified Dulbecco’s Medium at a 1:1 ratio, supplemented with 20% FBS and 0.05 mM 2-mercaptoethanol.

### Statistical analyses

The mean values with standard deviation (SD) or standard error of mean (SEM) were calculated using Prism 5 (GraphPad Software, San Diego, CA). Statistical comparisons between the indicated groups were made using two-tailed Student’s *t* test, one-way analysis of variance (ANOVA) and two-way ANOVA with Dunnett’s multiple comparisons test. *P* values < 0.05 were considered statistically significant for all experiments.

## Results

### In vitro sensitivity of FLT3-ITD AML cell lines and primary samples to p300/CBP inhibitors

**Fig. 1:**
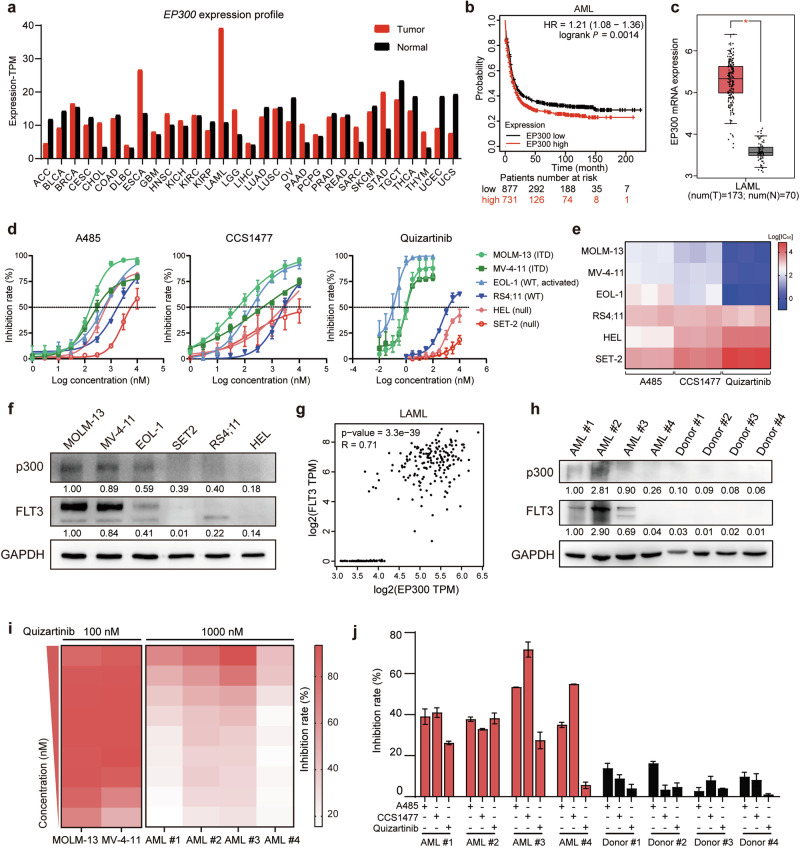
In vitro sensitivity of FLT3-ITD AML cell lines and primary samples to p300/CBP inhibitors. **a** Analysis of the E1A binding protein P300 (*EP300*) expression profile in various tumor samples and paired normal tissue samples via the GEPIA database. TPM, transcripts per million. **b** Kaplan-Meier plotter analysis of the correlation between *EP300* expression and overall survival in AML patients (HR, hazard ratio; *P* < 0.01). **c** *EP300* mRNA levels in AML patient samples and normal tissue samples by analyzing data from the TCGA and GTEx datasets in the GEPIA database. *P < 0.05. **d** Dose-response curves of AML cell lines (MOLM-13, MV-4-11, EOL-1, RS4;11, HEL and SET-2) after treatment with A485, CCS1477 and quizartinib for 72 h. **e** A heatmap displaying the log-transformed IC_50_ values of A485, CCS1477 and quizartinib across various AML cell lines from three independent replicates. The color spectrum ranged from dark blue, indicating the greatest sensitivity, to dark red, indicating the least sensitivity. **f** Western blot analysis was used to assess the protein expression of FLT3 and p300 in different AML cell lines, with GAPDH serving as a control. **g** Scatter plots were obtained from the GEPIA database to show the correlation between *EP300* and *FLT3* mRNA levels in AML, with R representing the Spearman correlation coefficient. **h** FLT3 and p300 protein expression in mononuclear cells isolated from four primary FLT3-ITD^+^ AML patient samples and four healthy donors. **i** Heatmaps of AML cell lines (MOLM-13 and MV-4-11) and primary FLT3-ITD^+^ AML patient samples after treatment with primary concentrations of 100 nM and 1000 nM quizartinib, respectively, in a twofold dilution manner for 5 days. **j** Mononuclear cells isolated from four primary FLT3-ITD^+^ AML patient samples and four healthy donors were treated with 125 nM A485, CCS1477 or quizartinib for 5 days, followed by cell counting kit-8 (CCK8) analysis for cell viability detection. The data are shown as means ± standard deviation (SD) from three independent experiments.

Firstly, we explored the role of p300/CBP in AML by analyzing the mRNA levels of *EP300* and *CREBBP* in patient samples from TCGA using the GEPIA database. The results revealed that the expression levels of *EP300* and *CREBBP* (red bar) were greater in LAML than in other cancer types (Fig. 1a; Supplementary Fig. S1a). High expression of *EP300* correlated with poorer OS through Kaplan-Meier plotter survival analysis (Fig. 1b). Furthermore, the expression of *EP300* and *CREBBP* in AML patient samples was significantly upregulated compared with that in paired normal samples (Fig. 1c; Supplementary Fig. S1b). Considering the role of FLT3 in AML, we evaluated the response of six representative AML cell lines with different genotypes at the *FLT3* locus to the treatment with p300/CBP inhibitors A485 or CCS1477. The results showed that p300/CBP inhibitors, similar to FLT3 inhibitors, exhibited lower IC_50_ values in two FLT3-ITD AML cell lines (MV-4-11 and MOLM-13) and a FLT3-activated cell line (EOL-1) than in the FLT3-WT (RS4;11) and FLT3 null cell lines (HEL and SET-2) (Fig. 1d, e; Supplementary Fig. S1c). Accordingly, A485 induced cell cycle arrest at the G_0_/G_1_ phase in the FLT3-ITD MV-4-11 cell line, but not in the FLT3-WT RS4;11 cell line or in the FLT3-null HEL cell line (Supplementary Fig. S1d, e). Further investigation revealed that FLT3-activated AML cell lines exhibited consistently high expression levels of FLT3 and p300 compared with those in FLT3 WT or null cells (Fig. 1f).

To extend this finding, we utilized the GEPIA database and observed a positive correlation between the expression of *EP300* and that of *FLT3* in LAML patients (Fig. 1g, *R* = 0.71, *P* < 0.001). Next, we investigated the expression of p300 and FLT3 in four patient-derived AML samples harboring FLT3-ITD mutation obtained at the time of diagnosis without prior treatment (patient information is provided in Table S3). Primary AML blasts exhibited consistently high expression levels of FLT3 and p300 compared to that in healthy donors (Fig. 1h). However, these AML patient samples unexpectedly exhibited lower sensitivity to the FLT3 inhibitor quizartinib compared to FLT3-ITD AML cell lines (Fig. 1i). While p300/CBP inhibitors exhibited significant cytotoxicity in these primary FLT3-ITD^+^ AML patient cells, they demonstrated minimal effects on PBMCs derived from healthy donors (Fig. 1j). Taken together, these results suggest that elevated p300 expression predicts poor prognosis in AML patients and that targeting p300/CBP is predominantly effective against AML cells with FLT3-ITD mutation.

### p300/CBP regulates FLT3 transcription through histone acetylation in FLT3-ITD AML cells

**Fig. 2:**
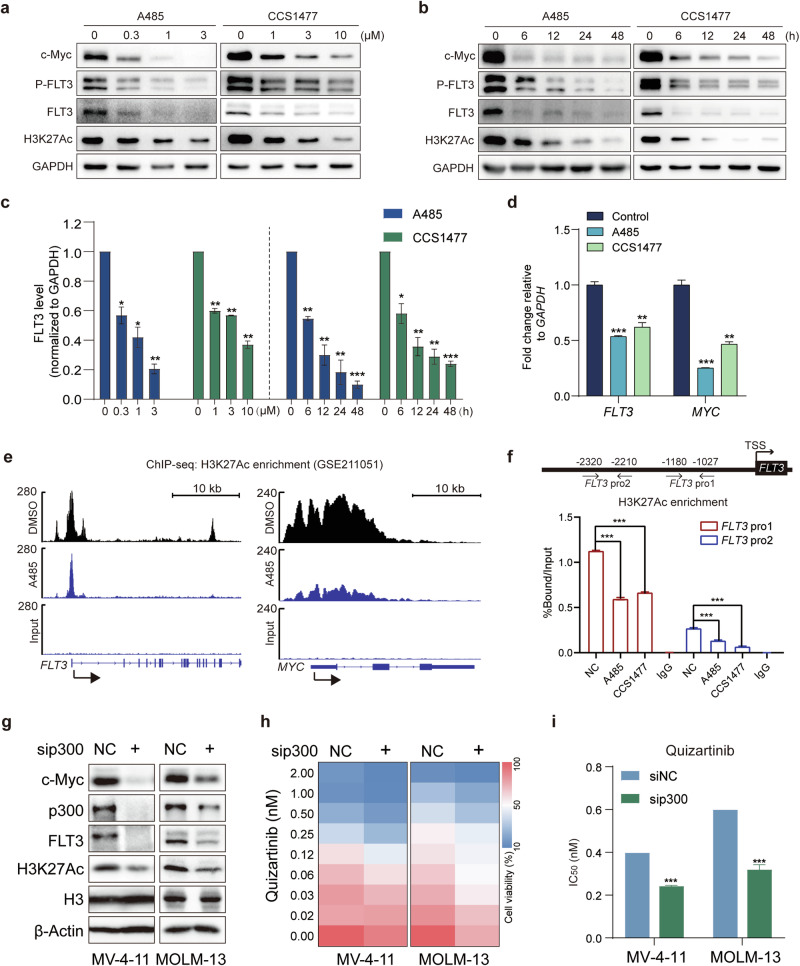
p300/CBP regulates FLT3 transcription through histone acetylation in FLT3-ITD AML cells. **a** Western blot analysis of MV-4-11 cells treated with the indicated doses of A485 or CCS1477 for 24 h. **b** Western blot analysis of MV-4-11 cells treated with 1 μM A485 or CCS1477 for the indicated durations. **c** Quantification of FLT3 protein expression relative to that of GAPDH in MV-4-11 cells treated with A485 and CCS1477 as described in **a** and **b**. **d** RT-qPCR assay for *FLT3* and *MYC* mRNA levels in MV-4-11 cells after treatment with 3 μM A485 or CCS1477 for 24 h. **e** Exemplar H3K27 acetylation (H3K27Ac) peaks at the *FLT3* and *MYC* loci in MOLM-13 cells treated with 3 μM A485 for 2 h, sourced from published ChIP-seq data in the GEO database (GSE211051). **f** ChIP-qPCR was used to analyze the enrichment of H3K27Ac at the *FLT3* promoter region in MV-4-11 cells after treatment with A485 (3 μM) or CCS1477 (5 μM) for 12 h. **g** Western blot analysis of p300, FLT3, c-Myc and H3K27Ac in sip300- or siNC-transfected MV-4-11 and MOLM-13 cells. **h** Heatmaps showing the proliferative inhibition of quizartinib in MV-4-11 and MOLM-13 cells transfected with sip300 or siNC from three independent experiments. **i** MV-4-11 and MOLM-13 cells transfected with siNC or sip300 were exposed to increasing concentrations of quizartinib for 72 h. MTT assays were performed to determine cell viability, and IC_50_ values were calculated using Prism 5. The data are presented as means ± SD from three independent experiments. *P < 0.05, **P < 0.01, ***P < 0.001 determined by Student’s *t* test.

Given the positive relationship between FLT3 and p300 expression, as well as the comparable sensitivities of their inhibitors toward AML cell lines, we conducted further analyses to ascertain whether the transcriptional activator p300 could directly regulate FLT3 transcription. After treatment with A485 or CCS1477 in MV-4-11 cells, the protein levels of FLT3 and its phosphorylated form decreased concomitantly in a dose- and time-dependent manner, along with the reduction of c-Myc and H3K27Ac, which are known targets of p300/CBP (Fig. 2a–c). RT-qPCR confirmed that A485 or CCS1477 treatment significantly inhibited the gene transcription of both *FLT3* and *MYC* in MV-4-11 cells (Fig. 2d). Previous reports have shown that CCS1477 treatment induces loss of the p300 signal, accompanied by the downregulation of H3K27Ac density at target gene loci [32]. In addition, ChIP-seq analysis of data from the GEO database (GSE211051) [38] demonstrated that MOLM-13 cells treated with A485 exhibited a distinct reduction in H3K27Ac enrichment at the gene promoters of *FLT3* and *MYC* (Fig. 2e). Consistent with the ChIP-seq results, compared with the control, A485 and CCS1477 induced a notable decrease in H3K27Ac recruitment to the promoter of *FLT3* in MV-4-11 cells (Fig. 2f). These results suggest that the transcriptional downregulation of *FLT3* induced by p300/CBP inhibitors treatment is associated with the abrogation of H3K27Ac abundance at the *FLT3* gene locus.

Moreover, transient silencing of p300 by siRNA in MV-4-11 and MOLM-13 cells significantly suppressed the protein levels of FLT3, H3K27Ac and c-Myc (Fig. 2g; Supplementary Fig. S2a). To elucidate the potential role of p300 in manipulating sensitivity to FLT3 inhibitors, we evaluated the efficacy of quizartinib in p300-knockdown MV-4-11 and MOLM-13 cells. Our results revealed that the cytotoxicity of quizartinib was substantially enhanced upon p300 depletion, resulting in an average twofold decrease in the IC_50_ value compared with that in siRNA control-treated cells (Fig. 2h, i). Taken together, these findings support the hypothesis that p300/CBP directly regulates FLT3 transcription by facilitating H3K27Ac recruitment to its promoter region, thereby accounting for the sensitivity of AML cells to p300/CBP inhibitors.

### The combination of p300/CBP inhibitors and quizartinib exhibits synergistic antileukemic activity through enhanced inhibition of FLT3 signaling and H3K27Ac

**Fig. 3:**
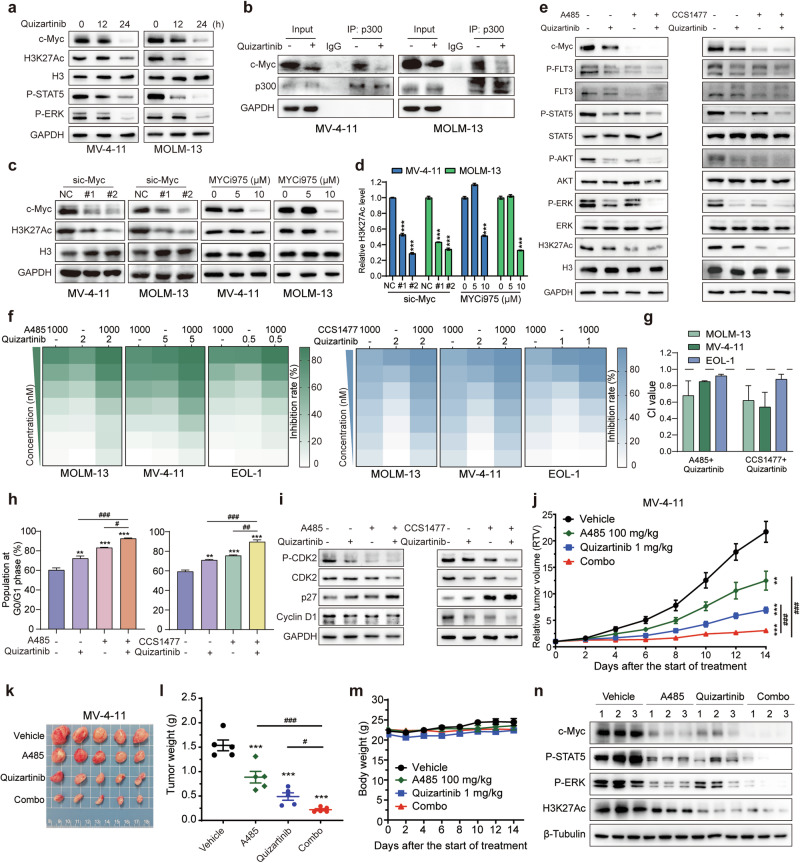
The combination of p300/CBP inhibitors and quizartinib exhibits synergistic antileukemic activity through enhanced inhibition of FLT3 signaling and H3K27Ac. **a** Western blot analyses assessed the levels of H3K27Ac, c-Myc, P-STAT5 and P-ERK in the MV-4-11 and MOLM-13 cell lines treated with 1 nM quizartinib for 12 or 24 h. **b** Lysates of MV-4-11 and MOLM-13 cells treated with or without 1 nM quizartinib for 12 h were immunoprecipitated with antibodies against p300 or the IgG control. The binding of endogenous c-Myc with p300 was examined by Western blotting. **c, d** MV-4-11 and MOLM-13 cells were transfected with siRNAs targeting c-Myc for 48 h or exposed to MYCi975 (5, 10 μM) for 24 h. H3K27Ac levels were analyzed by Western blotting and quantified by ImageJ. **e** FLT3 signaling pathways in MV-4-11 cells treated with quizartinib (1 nM), A485 (1 μM)/CCS1477 (3 μM), or their combination for 24 h were determined by Western blotting. **f** Heatmaps showing the proliferative inhibition of A485 or CCS1477 combined with quizartinib in FLT3-activated AML. AML cells were treated with primary concentrations of 1000 nM A485/CCS1477, as well as the indicated concentrations of quizartinib, in a twofold dilution manner for 72 h, followed by the assessment of cell viability using the MTT assay. **g** The combination index (CI) values of the combinations of p300/CBP inhibitors and quizartinib were calculated. **h** The cell cycle phase distributions of cells treated with quizartinib, A485, or CCS1477, either alone or in combination for 24 h were determined by flow cytometry. Cumulative percentages of cells in the G_0_/G_1_ phase are shown. The data are presented as means ± SD from three independent experiments. **i** Western blot analysis was conducted to assess the expression of cell cycle-related proteins in MV-4-11 cells treated with quizartinib (1 nM), A485/CCS1477 (500 nM), either alone or in combination for 24 h. Nude mice were engrafted with MV-4-11 cells, and mice (*n* = 5/group) were treated with vehicle, A485 (100 mg/kg), quizartinib (1 mg/kg), or their combination for 14 days. Tumor volumes **j**, tumor photos **k**, tumor weights **l**, and mouse weights **m** were measured at specific time points during the treatment period. The data are shown as means ± standard error of mean (SEM). **P < 0.01, ***P < 0.001 versus the vehicle; ^#^*P* < 0.05, ^##^*P* < 0.01, ^###^*P* < 0.001 versus either single agent alone. **n** The tumor tissues were subjected to Western blotting.

The relationship between FLT3-ITD and the epigenetic regulatory machinery has been increasingly elucidated in recent studies [39, 40]. A previous study demonstrated that knockdown of FLT3-ITD significantly reduced the enrichment of p300 and H3K27Ac at the promoter region of CHK1, suggesting that FLT3-ITD may recruit acetylation coactivators to promote target gene transcription [41]. Our results revealed that treatment with the FLT3-ITD inhibitor quizartinib resulted in a time-dependent attenuation of overall H3K27 acetylation, along with a reduction in downstream signaling of FLT3, including P-STAT5 and P-ERK in MV-4-11 and MOLM-13 cells (Fig. 3a; Supplementary Fig. S2b). Given that c-Myc has been reported to bind the coactivator p300 and promote gene transcription by regulating H3K27 modification [42, 43], the decline in H3K27Ac level observed with quizartinib treatment may be linked to the inhibition of c-Myc expression. Similarly, c-Myc strongly bound to p300 in both MV-4-11 and MOLM-13 cells, which was notably decreased following quizartinib treatment (Fig. 3b; Supplementary Fig. S2c). Furthermore, knockdown of c-Myc using siRNAs or inhibition of c-Myc via the small-molecule inhibitor MYCi975 significantly attenuated H3K27 acetylation (Fig. 3c, d; Supplementary Fig. S2d). These data imply that c-Myc forms a transcriptionally active complex with p300/CBP by promoting the acetylation of H3K27.

Although p300/CBP inhibitors have demonstrated selective activity against AML patients with FLT3-ITD mutation, their efficacy as monotherapy is limited, necessitating further investigation into the concurrent inhibition of p300/CBP and FLT3. Cotreatment with quizartinib and p300/CBP inhibitors resulted in a mutual suppression of FLT3 signaling and H3K27Ac, leading to greater reductions in the phosphorylation levels of STAT5, AKT, and ERK, as well as the expression of c-Myc (Fig. 3e; Supplementary Fig. S2e). Next, we further examined the potential cytotoxic synergism between p300/CBP inhibitors and quizartinib in the FLT3-activated cell lines, MOLM-13, MV-4-11 and EOL-1. As expected, the cell viability results demonstrated that the combination of A485 and quizartinib had synergistic antiproliferative effects on the FLT3-ITD cell lines MOLM-13 and MV-4-11, as well as on the FLT3-WT- activated cell line EOL-1, with CI values below 1 (Fig. 3f, g). Similar outcomes were observed with the combination of CCS1477 and quizartinib (Fig. 3f, g). Consistent with these findings, flow cytometry analysis revealed that the p300/CBP inhibitors A485 or CCS1477, similar to quizartinib, induced cell cycle G_0_/G_1_ phase arrest in MV-4-11 cells, which was enhanced when combined with quizartinib (Fig. 3h; Supplementary Fig. S3a, b). Cotreatment led to a synergistic reduction in the expression of G_1_ phase-related proteins, such as CDK2 and Cyclin D1, accompanied by the upregulation of p27, a crucial regulator that prevents cell entry into the S phase and controls cell proliferation [44] (Fig. 3i; Supplementary Fig. S3c). Moreover, the KEGG and GO pathway enrichment analyses of the ChIP-seq data from the GEO database (GSE211051) also revealed significant enrichment of the cell cycle, homologous combination and DNA replication upon treatment with A485 (Supplementary Fig. S3d, e).

Having observed synergistic effects in vitro, we proceeded to evaluate the combined antitumor efficacy of A485 and quizartinib in vivo in MV-4-11 xenograft models. Daily administration of 100 mg/kg A485 or 1 mg/kg quizartinib individually moderately impeded tumor growth, whereas their combination led to a greater reduction in tumor volumes and tumor weights (Fig. 3j–l). Moreover, the treatment was well tolerated throughout the experiment, as indicated by no significant body weight loss or mortality across all groups (Fig. 3m). In agreement with the in vitro results, coadministration of A485 and quizartinib enhanced the inhibition of H3K27Ac, c-Myc and FLT3 signaling, as evidenced by the reduced phosphorylation of STAT5 and ERK in tumor tissues (Fig. 3n; Supplementary Fig. S3f). Overall, p300/CBP inhibitors in combination with FLT3 inhibitors synergistically suppress p300/CBP targets and FLT3 signaling, resulting in augmented antitumor effects against FLT3-ITD AML both in vitro and in vivo.

### The p300/CBP-related gene transcription profile is associated with quizartinib resistance

**Fig. 4:**
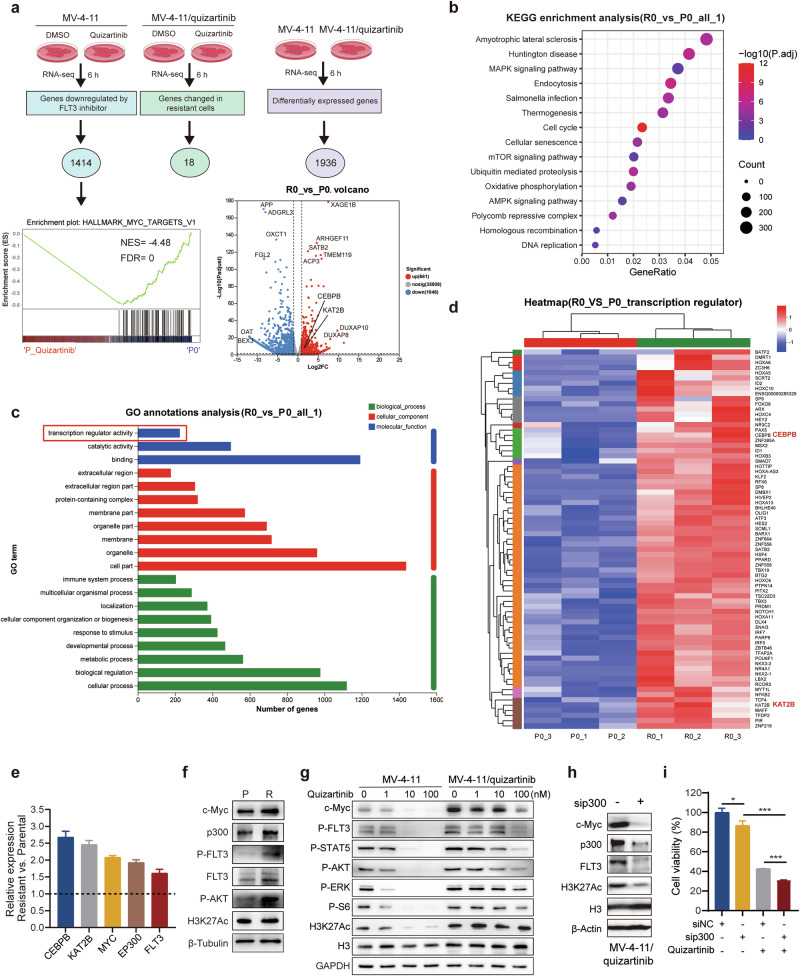
Differential expression profiles of genes associated with p300/CBP in MV-4-11 and MV-4-11/quizartinib cells. **a** Schematic diagram of the integrated transcription genome profiling of MV-4-11 parental (P) cells and MV-4-11/quizartinib (R) cells treated with DMSO or quizartinib (20 nM) for 6 h. GSEA of MYC targets in MV-4-11 DMSO- versus quizartinib-treated cells, and volcano plots of DEGs in resistant cells versus parental cells are shown. **b** KEGG enrichment analysis between MV-4-11/quizartinib cells and MV-4-11 cells using RNA-seq data. **c** GO analysis of molecular function, cellular component and biological process terms comparing MV-4-11/quizartinib and MV-4-11 cells revealed that transcription regulator activity was one of the top-ranking categories. **d** Gene cluster analysis of genes related to transcription regulator activity via GO analysis. **e** mRNA levels of *CEBPB*, *KAT2B*, *EP300*, *FLT3*, and *MYC* in MV-4-11/quizartinib cells compared to parental MV-4-11 cells were detected by RT-qPCR. **f** Western blots were performed to assess the expression of c-Myc, p300, FLT3 and H3K27Ac in MV-4-11 and MV-4-11/quizartinib cells. **g** The FLT3 signaling pathway in parental and resistant cells treated with increasing concentrations of quizartinib for 24 h was detected by Western blotting. **h** Western blots were conducted to evaluate p300, FLT3, c-Myc and H3K27Ac protein expression in MV-4-11/quizartinib cells transfected with either siNC or sip300. **i** Cell viability of siNC- or sip300-transfected MV-4-11/quizartinib cells following quizartinib (200 nM) treatment for 72 h was assessed by the MTT assay. The data are presented as means ± SD from three independent experiments. *P < 0.05, ***P < 0.001.

Patients frequently develop resistance and commonly experience clinical relapse following extended monotherapy with FLT3 inhibitors. To investigate the possible role of p300/CBP in conferring resistance to quizartinib, we utilized an MV-4-11/quizartinib cell line with a FLT3-ITD/F691L mutation, which was previously established and exhibited resistance to quizartinib [45, 46]. The sensitivity of MV-4-11/quizartinib cells to quizartinib was approximately 200-fold lower than that of parental MV-4-11 cells (Table S4). We characterized the transcriptome alterations in MV-4-11 and MV-4-11/quizartinib cells following treatment with quizartinib or DMSO for 6 h by RNA-seq analysis (Fig. 4a; Supplementary Fig. S4a). In parental MV-4-11 cells, a total of 1414 transcripts were significantly downregulated by quizartinib. Gene set enrichment analysis (GSEA) revealed that the MYC-target gene set was downregulated in quizartinib-treated MV-4-11 cells. In contrast, quizartinib did not cause such significant transcriptomic changes in MV-4-11/quizartinib cells. Moreover, a total of 1936 differentially expressed genes (DEGs, *P*_adjust < 0.05, fold change ≥ 2 or fold change ≤ 0.5) were identified in MV-4-11/quizartinib cells compared with MV-4-11 cells, among which 770 were upregulated and 1166 were downregulated (Fig. 4a; Supplementary Fig. S4b). KEGG enrichment analysis revealed that these DEGs were enriched in several pathways, including the cell cycle, MAPK signaling, mTOR signaling and DNA replication (Fig. 4b). GO enrichment analysis indicated that one of the significantly enriched gene sets was related to transcription regulator activity (Fig. 4c). When subjected to KEGG analysis, these genes were associated with TGF-β, cell cycle, Wnt and Notch signaling pathways (Supplementary Fig. S4c). Gene cluster analysis further revealed that all these transcripts were upregulated in MV-4-11/quizartinib cells, including *KAT2B*, a homologous gene of *EP300*, and *CEBPB*, a known transcriptional cofactor of *EP300* (Fig. 4d). Consistent with the RNA-seq results, compared with parental cells, quizartinib-resistant cells exhibited notable increases of *KAT2B* and *CEBPB* mRNA levels, along with enhanced expression of *EP300*, *FLT3*, and *MYC*, as determined by RT-qPCR analysis (Fig. 4e). The elevated levels of c-Myc, p300, FLT3 and H3K27Ac in MV-4-11/quizartinib cells were also confirmed by Western blot analysis (Fig. 4f; Supplementary Fig. S4d). These data suggest that p300/CBP and its related transcripts may be associated with resistance to quizartinib in FLT3-ITD AML cells.

We further validated the role of p300/CBP in mediating resistance to FLT3 inhibitors. In the quizartinib-resistant cell line, quizartinib exhibited limited inhibition of FLT3 signaling, H3K27Ac, and c-Myc, contrasting with its effects on parental cells (Fig. 4g; Supplementary Fig. S4e). In addition, knockdown of p300 in MV-4-11/quizartinib cells led to a marked reduction in FLT3, H3K27Ac and c-Myc levels (Fig. 4h; Supplementary Fig. S4f), which in turn sensitized MV-4-11/quizartinib cells to quizartinib treatment (Fig. 4i). Overall, these findings suggest that p300/CBP and its cofactors are highly expressed in MV-4-11/quizartinib cells, potentially contributing to FLT3 inhibitor resistance by regulating FLT3 and c-Myc expression.

### The combination of p300/CBP inhibitors and quizartinib overcomes acquired resistance to FLT3 inhibitors both in vitro and in vivo

**Fig. 5:**
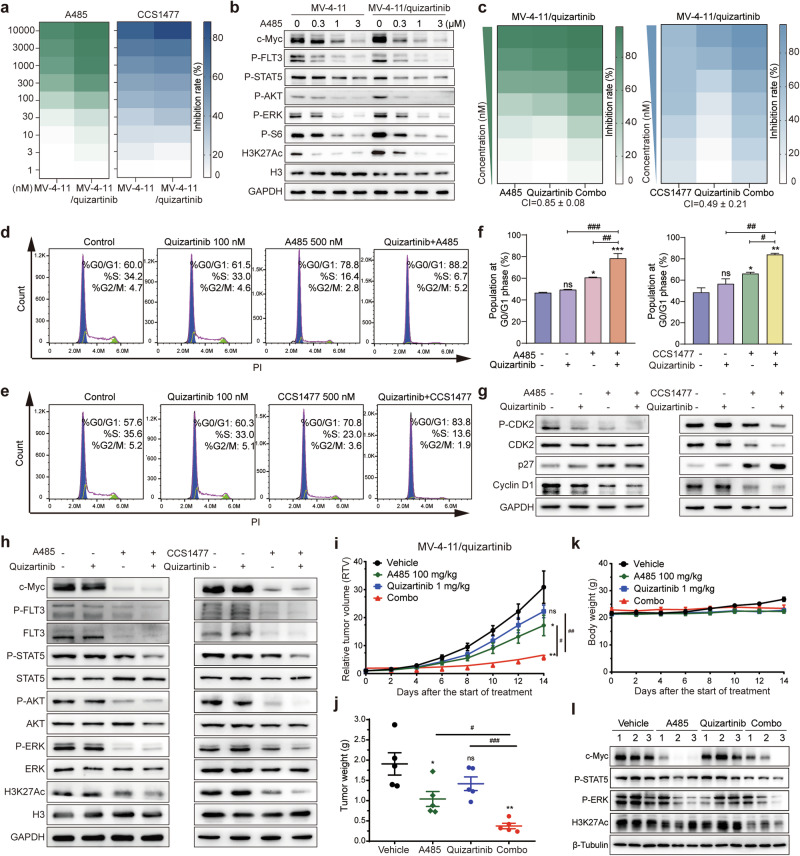
The combination of p300/CBP inhibitors and quizartinib overcomes acquired resistance to FLT3 inhibitors both in vitro and in vivo. **a** Heatmap showing the cytotoxicity of A485 and CCS1477 in MV-4-11 parental and MV-4-11/quizartinib resistant cells using the MTT assay. **b** Western blots were performed on MV-4-11 parental and resistant cells treated with increasing concentrations of A485 for 24 h. **c** Heatmap showing the proliferative inhibition of p300/CBP inhibitors combined with quizartinib on MV-4-11/quizartinib cells. MV-4-11/quizartinib cells were treated with primary concentrations of A485/CCS1477 (1 μM) or quizartinib (1 μM), either alone or in combination in a twofold dilution manner for 72 h. CI values were calculated and shown. Cell cycle phase distributions of cells treated with quizartinib (100 nM), A485 **d** /CCS1477 **e** (500 nM), either alone or in combination, for 24 h were determined by flow cytometry. **f** Cumulative percentages of cells in the G_0_/G_1_ phase following monotreatment or combination treatment in **d** and **e** were shown. The data are shown as means ± SD from three independent experiments. **g** Western blot analysis was conducted to assess G_1_ phase-related proteins in MV-4-11/quizartinib cells treated with quizartinib (100 nM), A485/CCS1477 (500 nM), either alone or in combination, for 24 h. **h** The FLT3 signaling pathway was assessed in MV-4-11/quizartinib cells treated with quizartinib (10 nM), A485 (1 μM), or CCS1477 (3 μM) along or in combination for 24 h by Western blotting. Nude mice were engrafted with MV-4-11/quizartinib cells, and the mice (*n* = 5/group) were treated with vehicle, A485 (100 mg/kg), quizartinib (1 mg/kg), or their combination for 14 days. Tumor volumes **i**, tumor weights **j**, and mouse weights **k** were measured at specified time points during the treatment period. The data are shown as means ± SEM. *P < 0.05, **P < 0.01, ***P < 0.001 versus the vehicle; ^#^*P* < 0.05, ^##^*P* < 0.01, ^###^*P* < 0.001 versus either single agent alone. **l** Related protein expression in tumor tissues was detected by Western blotting.

To explore whether targeting p300/CBP could overcome resistance to quizartinib, we evaluated the activity of the p300/CBP inhibitors A485 and CCS1477, along with the chemotherapeutic drug paclitaxel as a reference. We found that these agents exhibited similar efficacy in MV-4-11-resistant cell lines compared to parental cells (Fig. 5a; Supplementary Fig. S5a, b). Furthermore, A485 caused G_0_/G_1_ phase arrest in both cell lines, whereas quizartinib exerted marked effects only on MV-4-11 parental cells not on resistant cells (Supplementary Fig. S5c, d). In addition, A485, unlike quizartinib, significantly inhibited FLT3 signaling, H3K27Ac, and c-Myc in MV-4-11/quizartinib cells, akin to its effect on MV-4-11 parental cells (Fig. 5b; Supplementary Fig. S5e).

We then investigated whether concurrent inhibition of p300/CBP and FLT3 could enhance therapeutic efficacy in FLT3 inhibitor-resistant models. Cell proliferation assay revealed synergistic effects of A485 or CCS1477 in combination with quizartinib on MV-4-11/quizartinib cells, as evidenced by CI values less than 1 (Fig. 5c). Additionally, we examined the impact of combined treatment with A485/CCS1477 and quizartinib on the cell cycle. Flow cytometry analysis of MV-4-11/quizartinib cells revealed a marked increase in the percentage of cells at the G_0_/G_1_ phase in the combination group compared with that in the monotreated groups (Fig. 5d–f). Consistently, there was a notable decrease in the expression of G1 phase-related proteins (Fig. 5g; Supplementary Fig. S5f), as well as reductions in FLT3 signaling and H3K27Ac (Fig. 5h; Supplementary Fig. S5g) in the cotreatment group.

We subsequently established MV-4-11/quizartinib xenograft models to further evaluate the efficacy of combined treatment with A485 and quizartinib in vivo. As expected, monotherapy with quizartinib showed limited efficacy in MV-4-11/quizartinib xenograft models; however, when combined with A485, there was a significant enhancement in antitumor efficacy (Fig. 5i, j; Supplementary Fig. S5h). Importantly, no significant body weight loss was observed in any of the treatment groups, indicating a favorable safety profile (Fig. 5k). Additionally, the coadministration of A485 and quizartinib enhanced the inhibition of H3K27Ac and c-Myc expression, as well as FLT3 downstream signaling, including STAT5 and ERK phosphorylation, in tumor tissues from MV-4-11/quizartinib xenografts (Fig. 5l; Supplementary Fig. S5i). Taken together, these results suggest that p300/CBP inhibitors preserve efficacy in FLT3 inhibitor-resistant AML models harboring TKD mutations and demonstrate a synergistic effect when combined with a FLT3 inhibitor.

### The combination of p300/CBP inhibitors and quizartinib has synergistic effects on primary FLT3-ITD^+^ AML samples

**Fig. 6:**
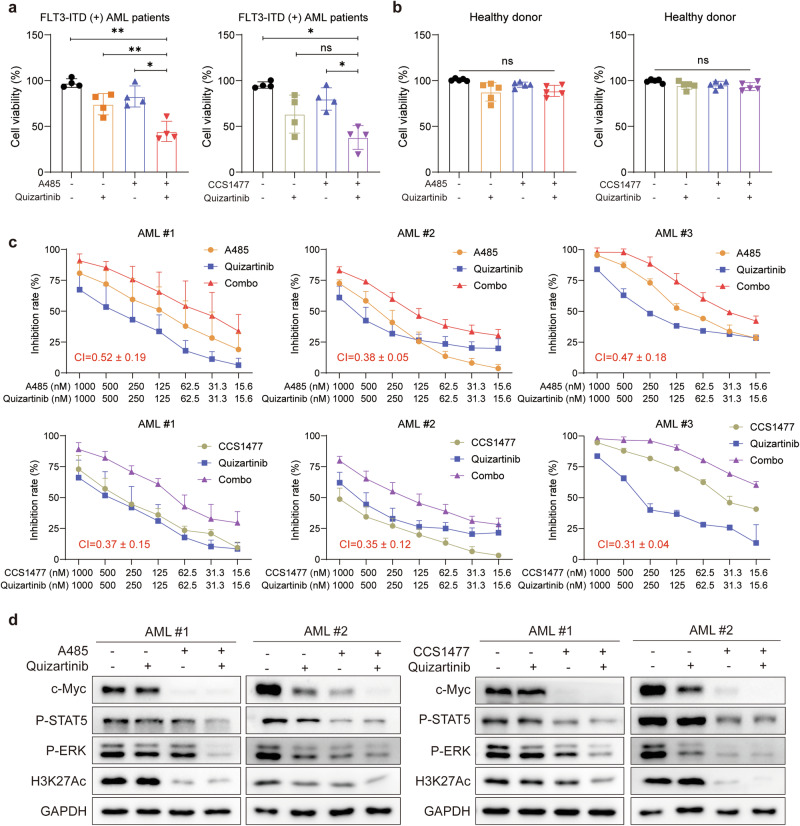
The combination of p300/CBP inhibitors and quizartinib has synergistic effects on primary FLT3-ITD^+^ AML samples. Cell viability of four primary FLT3-ITD^+^ AML samples **a** or peripheral blood mononuclear cells from five healthy donors **b** was assessed after exposure to 62.5 nM A485/CCS1477, quizartinib alone, or in combination for 5 days. The data are shown as means ± SD from three independent experiments. *P < 0.05, **P < 0.01, ns indicates not significant, as determined by Student’s *t* test. **c** Primary FLT3-ITD^+^ patient samples (#1, #2, #3) were treated with the indicated doses of A485/CCS1477, quizartinib, or their combination for 5 days, followed by cell viability assessment using the CCK8 assay and calculation of CI values. The data are shown as means ± SEM from at least three biological replicates. **d** Western blots were performed on primary FLT3-ITD^+^ patient samples (#1, #2) treated with 1 μM quizartinib, A485/CCS1477, either alone or in combination for 24 h. FLT3 signaling, c-Myc and H3K27Ac were detected with GAPDH used as a loading control.

To further evaluate the possible clinical benefit of the combination strategy, we examined the efficacy of cotreating primary AML cells from patients with varying FLT3-ITD allelic ratios with p300/CBP inhibitors and quizartinib (patient information is provided in Table S3). Consistent with our aforementioned findings, all the primary AML samples exhibited a noteworthy reduction in the number of viable cells with the combination treatment compared with either monotherapy (Fig. 6a). Additionally, treatment of PBMCs isolated from healthy donors with the same doses of drugs revealed no significant effect on cell viability, indicating minimal nonspecific toxicity in healthy individuals (Fig. 6b). Notably, the cytotoxic effects of p300/CBP inhibitors on the FLT3-ITD^+^ AML patient samples were comparable to those observed in the AML cell lines MV-4-11 and MOLM-13, whereas quizartinib exhibited relatively weaker activity in the AML patient samples (Fig. 6c). Furthermore, the combination of p300/CBP inhibitors and quizartinib had synergistic effects on these FLT3-ITD^+^ AML samples, as evidenced by markedly low CI values ranging from 0.30 to 0.52 (Fig. 6c). Consistent with these results, quizartinib had a limited inhibitory effect on P-STAT5, P-ERK, H3K27Ac and c-Myc in FLT3-ITD^+^ primary samples, whereas these signaling effectors were significantly suppressed when combined with either A485 or CCS1477 treatment (Fig. 6d; Supplementary Fig. S6a, b). Overall, these data obtained from primary FLT3-ITD^+^ AML patient samples further substantiate that the combination of p300/CBP inhibitors and FLT3 inhibitors concomitantly suppresses both FLT3 signaling and H3K27Ac, demonstrating synergistic antileukemia effects and highlighting considerable promise for the clinical application of such combination therapies.

## Discussion

**Fig. 7:**
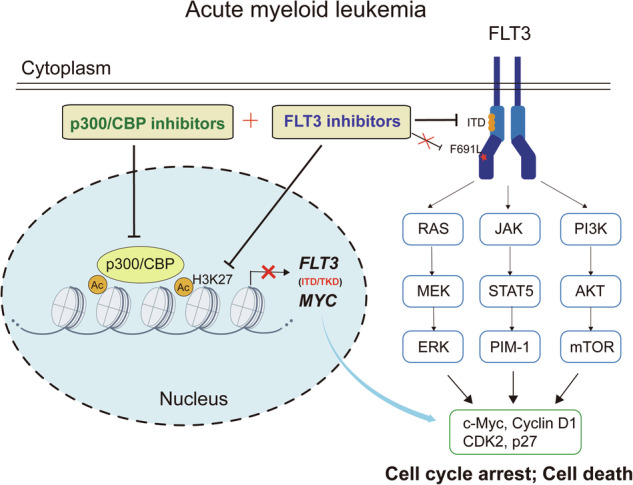
Schematic illustration of the synergistic effects of cotargeting p300/CBP and FLT3 in AML. In AML carrying the FLT3 mutation, p300/CBP regulates the transcription of FLT3 and MYC by modulating H3K27Ac at the gene loci. Coinhibition of p300/CBP and FLT3 synergistically suppresses FLT3 signaling and H3K27Ac, leading to cell death and overcoming resistance both in xenograft models and in primary AML patient samples.

FLT3-ITD mutation is a common driver mutation associated with a high leukemic burden and poor prognosis in AML patients. Despite the potent and selective activities of FLT3 inhibitors, resistance frequently emerges following prolonged exposure, often accompanied by secondary mutations in FLT3-ITD or TKD, presenting a significant challenge [47]. p300/CBP regulates gene transcription by modulating histone acetylation, predominantly affecting substrates crucial for hematopoietic maintenance and development [48, 49]. The overexpression of p300/CBP is associated with cancer progression and poor prognosis, and p300/CBP inhibitors have shown significant promise in suppressing hematopoietic malignancies [49]. However, the role of p300/CBP remains inadequately studied in FLT3-ITD AML, especially in the context of R/R FLT3-ITD AML. In this study, we demonstrated that p300/CBP inhibitors predominantly inhibited the proliferation of FLT3-ITD AML cells, effectively overcame resistance to FLT3 inhibitors in both cell lines and primary AML patients, and augmented sensitivity to FLT3 inhibitors in both in vitro and in vivo models (Fig. 7).

As previously demonstrated, p300 plays an important role in AML progression and drug resistance [40, 50]. Analysis of the GEPIA database indicated that high expression of p300 was associated with unfavorable OS in patients and was positively correlated with FLT3 in AML patients. Consistent with these findings, we also found that both FLT3-ITD AML cell lines and primary AML blasts exhibited elevated expression levels of FLT3 and p300. The p300/CBP complex serves as a histone acetyltransferase that primarily catalyzes H3K27 acetylation [51] while also possessing the ability to acetylate nonhistone substrates. Additionally, it engages in multiple interactions with transcription factors, chromatin remodeling complexes, and the basal transcriptional machinery [52]. In this study, we further revealed that p300 directly regulated FLT3 transcription through facilitating H3K27Ac recruitment at its gene locus. Consequently, p300/CBP inhibitors such as A485 and CCS1477 predominantly induced cell cycle arrest and exhibited enhanced cytotoxicity against AML cell lines harboring the FLT3-ITD mutation compared with those with WT or null FLT3. Moreover, p300/CBP inhibitors showed comparable efficacy in primary FLT3-ITD^+^ AML patient samples, while demonstrating minimal effect towards normal PBMCs from healthy donors. These findings indicate that the overexpression of p300 may predict poor OS in FLT3-ITD AML, highlighting its targeted therapy as a potentially curative option.

Numerous ongoing studies are currently evaluating the efficacy of combining diverse agents that target key signaling pathways through distinct mechanisms [53]. Both previous studies [54] and our current studies have shown that FLT3 inhibitors effectively suppress FLT3 activation and the phosphorylation of downstream effectors, such as STAT5, AKT, and ERK. In addition, it has been reported that FLT3-ITD increases CHK1 transcription by recruiting p300 and increasing H3K27Ac abundance at the CHK1 promoter in AML [41], indicating a transcriptional coactivator role of FLT3-ITD and its potential association with p300. c-Myc has been reported to directly bind and recruit p300 to the promoter regions of target genes, inducing transcription by H3K27Ac enhancement [42, 43]. Consistently, we confirmed in our study that in FLT3-ITD AML cells, c-Myc formed a complex with p300 and inhibition of c-Myc attenuated the overall level of H3K27 acetylation. Thus, the FLT3 inhibitor quizartinib was observed to significantly reduce H3K27Ac level, accompanied by downregulation of c-Myc in sensitive FLT3-ITD AML cell lines. Reciprocally, our results demonstrated that p300 promoted FLT3 transcription through histone acetylation and inhibition of p300/CBP suppressed FLT3 expression and downstream pathways, leading to selective efficacy in FLT3-ITD AML. Consequently, simultaneous inhibition of FLT3 and p300/CBP led to enhanced suppression of both FLT3 signaling and H3K27 acetylation, resulting in intensified G_0_/G_1_ phase cell cycle arrest, thereby demonstrating synergistic effects in AML cell lines and xenograft models. Management of FLT3-mutated AML typically involves standard treatment guidelines combining chemotherapy and FLT3 inhibitors, followed by allogeneic hematopoietic cell transplantation to improve the likelihood of promising outcomes [11]. Our study suggests that targeting both epigenetic and receptor tyrosine kinase signaling could serve as an alternative therapeutic strategy for treating FLT3-ITD AML. Further investigation into the underlying mechanism for their synergism is warranted.

FLT3 inhibitors have demonstrated less-than-expected effectiveness in R/R AML due to the emergence of resistance, including selective next-generation FLT3 inhibitors [11, 55]. The survival rates of elderly AML patients, particularly those aged 60 and above, remain unsatisfactory. A high allelic ratio (> 0.5) of FLT3-ITD is a driver mutation associated with a high leukemic burden, leading to poor prognosis and significantly affecting the management of AML patients [56]. In our study, quizartinib monotreatment exhibited limited efficacy both in resistant FLT3-ITD AML cells carrying the F691L mutation and in FLT3-ITD^+^ AML patient samples, particularly in older individuals with high allelic ratios, unlike its significant effectiveness in FLT3-ITD AML cell lines. In these models with resistance to quizartinib, p300/CBP inhibitors exhibited sustained potency and enhanced efficacy when combined with FLT3 inhibitors. Research has identified c-Myc, a key oncogenic transcription factor regulated by p300/CBP-mediated high-density of H3K27Ac at super enhancer regions, as being associated with AML progression and drug resistance [57]. Indeed, the p300/CBP inhibitors A485 and CCS1477 robustly decreased H3K27Ac and c-Myc expression in both quizartinib-resistant FLT3-ITD AML cell lines with TKD mutations and primary AML patient samples. Furthermore, the combination of p300/CBP inhibitors and quizartinib enhanced the inhibition of downstream pathways including FLT3/STAT5/ERK signaling, c-Myc and H3K27Ac. These encouraging findings of the significant synergism between p300/CBP inhibitors and FLT3 inhibitors establish a solid foundation for future clinical development. Subsequent research will incorporate FLT3-mutated R/R patient samples and patient-derived xenograft (PDX) models, including those with ITD/TKD mutations, to evaluate this combination strategy and explore additional potential drug combinations aimed at overcoming drug resistance.

In conclusion, our study demonstrates that p300/CBP inhibitors exhibit specific efficacy in FLT3-ITD AML, maintaining efficacy even in cases resistant to FLT3 inhibitors with FLT3-ITD/TKD mutations or primary AML samples with high ITD allelic ratios. The combination of p300/CBP inhibitors and the FLT3 inhibitor quizartinib leads to the mutual suppression of H3K27Ac and FLT3 signaling, resulting in synergistic antitumor effects in in vitro and in vivo models of FLT3-ITD AML. Taken together, our findings shed light on the potential of concurrently targeting both epigenetic factors and receptor tyrosine kinase signaling as a promising therapeutic approach for treating FLT3-ITD AML, particularly in R/R patients.

## Supplementary information

Supplementary Figure legends

Supplementary Figure S1

Supplementary Figure S2

Supplementary Figure S3

Supplementary Figure S4

Supplementary Figure S5

Supplementary Figure S6

Supplementary Tables

## Data Availability

All the data presented in this study are included in this published article and its additional files. Any data in this study are available from the corresponding author upon reasonable request.

## References

[1] Döhner H, Weisdorf DJ, Bloomfield CD. Acute myeloid leukemia. N Engl J Med. 2015;373:1136–52. 26376137 10.1056/NEJMra1406184

[2] Shimony S, Stahl M, Stone RM. Acute myeloid leukemia: 2023 update on diagnosis, risk-stratification, and management. Am J Hematol. 2023;98:502–26. 36594187 10.1002/ajh.26822

[3] Döhner H, Wei AH, Appelbaum FR, Craddock C, DiNardo CD, Dombret H, et al. Diagnosis and management of AML in adults: 2022 recommendations from an international expert panel on behalf of the ELN. Blood. 2022;140:1345–77. 35797463 10.1182/blood.2022016867

[4] Mrózek K, Kohlschmidt J, Blachly JS, Nicolet D, Carroll AJ, Archer KJ, et al. Outcome prediction by the 2022 European LeukemiaNet genetic-risk classification for adults with acute myeloid leukemia: an Alliance study. Leukemia. 2023;37:788–98. 36823396 10.1038/s41375-023-01846-8 PMC10079544

[5] Kazi JU, Rönnstrand L. FMS-like tyrosine kinase 3/FLT3: from basic science to clinical implications. Physiol Rev. 2019;99:1433–66. 31066629 10.1152/physrev.00029.2018

[6] Brasel K, Escobar S, Anderberg R, de Vries P, Gruss HJ, Lyman SD. Expression of the flt3 receptor and its ligand on hematopoietic cells. Leukemia. 1995;9:1212–8. 7630197

[7] Papaemmanuil E, Gerstung M, Bullinger L, Gaidzik VI, Paschka P, Roberts ND, et al. Genomic classification and prognosis in acute myeloid leukemia. N Engl J Med. 2016;374:2209–21. 27276561 10.1056/NEJMoa1516192 PMC4979995

[8] Sasaki K, Ravandi F, Kadia TM, DiNardo CD, Short NJ, Borthakur G, et al. De novo acute myeloid leukemia: a population-based study of outcome in the United States based on the Surveillance, Epidemiology, and End Results (SEER) database, 1980 to 2017. Cancer. 2021;127:2049–61. 33818756 10.1002/cncr.33458 PMC11826308

[9] Shallis RM, Wang R, Davidoff A, Ma X, Zeidan AM. Epidemiology of acute myeloid leukemia: recent progress and enduring challenges. Blood Rev. 2019;36:70–87. 31101526 10.1016/j.blre.2019.04.005

[10] Capelli D, Menotti D, Fiorentini A, Saraceni F, Olivieri A. Overcoming resistance: FLT3 inhibitors past, present, future and the challenge of cure. Cancers. 2022;14:4315. 36077850 10.3390/cancers14174315 PMC9454516

[11] Daver N, Schlenk RF, Russell NH, Levis MJ. Targeting FLT3 mutations in AML: review of current knowledge and evidence. Leukemia. 2019;33:299–312. 30651634 10.1038/s41375-018-0357-9 PMC6365380

[12] Killock D. Newly diagnosed AML: quizartinib improves OS. Nat Rev Clin Oncol. 2023;20:504. 37253972 10.1038/s41571-023-00787-6

[13] Perl AE, Martinelli G, Cortes JE, Neubauer A, Berman E, Paolini S, et al. Gilteritinib or chemotherapy for relapsed or refractory FLT3-mutated AML. N Engl J Med. 2019;381:1728–40. 31665578 10.1056/NEJMoa1902688

[14] Daver N, Venugopal S, Ravandi F. FLT3 mutated acute myeloid leukemia: 2021 treatment algorithm. Blood Cancer J. 2021;11:104. 34045454 10.1038/s41408-021-00495-3 PMC8159924

[15] Ambinder AJ, Levis M. Potential targeting of FLT3 acute myeloid leukemia. Haematologica. 2021;106:671–81. 32703795 10.3324/haematol.2019.240754 PMC7927884

[16] Leischner H, Albers C, Grundler R, Razumovskaya E, Spiekermann K, Bohlander S, et al. SRC is a signaling mediator in FLT3-ITD- but not in FLT3-TKD-positive AML. Blood. 2012;119:4026–33. 22411868 10.1182/blood-2011-07-365726

[17] Choudhary C, Schwäble J, Brandts C, Tickenbrock L, Sargin B, Kindler T, et al. AML-associated Flt3 kinase domain mutations show signal transduction differences compared with Flt3 ITD mutations. Blood. 2005;106:265–73. 15769897 10.1182/blood-2004-07-2942

[18] Sato T, Yang X, Knapper S, White P, Smith BD, Galkin S, et al. FLT3 ligand impedes the efficacy of FLT3 inhibitors in vitro and in vivo. Blood. 2011;117:3286–93. 21263155 10.1182/blood-2010-01-266742 PMC3069670

[19] Short NJ, Nguyen D, Ravandi F. Treatment of older adults with FLT3-mutated AML: Emerging paradigms and the role of frontline FLT3 inhibitors. Blood Cancer J. 2023;13:142. 37696819 10.1038/s41408-023-00911-w PMC10495326

[20] Zarrinkar PP, Gunawardane RN, Cramer MD, Gardner MF, Brigham D, Belli B, et al. AC220 is a uniquely potent and selective inhibitor of FLT3 for the treatment of acute myeloid leukemia (AML). Blood. 2009;114:2984–92. 19654408 10.1182/blood-2009-05-222034 PMC2756206

[21] Stelmach P, Trumpp A. Leukemic stem cells and therapy resistance in acute myeloid leukemia. Haematologica. 2023;108:353–66. 36722405 10.3324/haematol.2022.280800 PMC9890038

[22] McMahon CM, Ferng T, Canaani J, Wang ES, Morrissette JJD, Eastburn DJ, et al. Clonal selection with RAS pathway activation mediates secondary clinical resistance to selective FLT3 inhibition in acute myeloid leukemia. Cancer Discov. 2019;9:1050–63. 31088841 10.1158/2159-8290.CD-18-1453 PMC11994087

[23] Wang P, Xiao X, Zhang Y, Zhang B, Li D, Liu M, et al. A dual inhibitor overcomes drug-resistant FLT3-ITD acute myeloid leukemia. J Hematol Oncol. 2021;14:105. 34217323 10.1186/s13045-021-01098-y PMC8255005

[24] Lee HK, Kim HW, Lee IY, Lee J, Lee J, Jung DS, et al. G-749, a novel FLT3 kinase inhibitor, can overcome drug resistance for the treatment of acute myeloid leukemia. Blood. 2014;123:2209–19. 24532805 10.1182/blood-2013-04-493916 PMC3975259

[25] Xu B, Zhao Y, Wang X, Gong P, Ge W. MZH29 is a novel potent inhibitor that overcomes drug resistance FLT3 mutations in acute myeloid leukemia. Leukemia. 2017;31:913–21. 27773927 10.1038/leu.2016.297

[26] Larrosa-Garcia M, Baer MR. FLT3 inhibitors in acute myeloid leukemia: current status and future directions. Mol Cancer Ther. 2017;16:991–1001. 28576946 10.1158/1535-7163.MCT-16-0876 PMC5600895

[27] Chen Q, Yang B, Liu X, Zhang XD, Zhang L, Liu T, et al. Histone acetyltransferases CBP/p300 in tumorigenesis and CBP/p300 inhibitors as promising novel anticancer agents. Theranostics. 2022;12:4935–48. 35836809 10.7150/thno.73223 PMC9274749

[28] Delvecchio M, Gaucher J, Aguilar-Gurrieri C, Ortega E, Panne D. Structure of the p300 catalytic core and implications for chromatin targeting and HAT regulation. Nat Struct Mol Biol. 2013;20:1040–6. 23934153 10.1038/nsmb.2642

[29] He ZX, Wei BF, Zhang X, Gong YP, Ma LY, Zhao W. Current development of CBP/p300 inhibitors in the last decade. Eur J Med Chem. 2021;209:112861. 33045661 10.1016/j.ejmech.2020.112861

[30] Arif M, Pradhan SK, Thanuja GR, Vedamurthy BM, Agrawal S, Dasgupta D, et al. Mechanism of p300 specific histone acetyltransferase inhibition by small molecules. J Med Chem. 2009;52:267–77. 19086895 10.1021/jm800657z

[31] Lau OD, Kundu TK, Soccio RE, Ait-Si-Ali S, Khalil EM, Vassilev A, et al. HATs off: selective synthetic inhibitors of the histone acetyltransferases p300 and PCAF. Mol Cell. 2000;5:589–95. 10882143 10.1016/s1097-2765(00)80452-9

[32] Nicosia L, Spencer GJ, Brooks N, Amaral FMR, Basma NJ, Chadwick JA, et al. Therapeutic targeting of EP300/CBP by bromodomain inhibition in hematologic malignancies. Cancer Cell. 2023;41:2136–53.e13. 37995682 10.1016/j.ccell.2023.11.001

[33] Hay DA, Fedorov O, Martin S, Singleton DC, Tallant C, Wells C, et al. Discovery and optimization of small-molecule ligands for the CBP/p300 bromodomains. J Am Chem Soc. 2014;136:9308–19. 24946055 10.1021/ja412434f PMC4183655

[34] Rooney TP, Filippakopoulos P, Fedorov O, Picaud S, Cortopassi WA, Hay DA, et al. A series of potent CREBBP bromodomain ligands reveals an induced-fit pocket stabilized by a cation-π interaction. Angew Chem. 2014;53:6126–30. 24821300 10.1002/anie.201402750 PMC4298791

[35] Lasko LM, Jakob CG, Edalji RP, Qiu W, Montgomery D, Digiammarino EL, et al. Discovery of a selective catalytic p300/CBP inhibitor that targets lineage-specific tumours. Nature. 2017;550:128–32. 28953875 10.1038/nature24028 PMC6050590

[36] Welti J, Sharp A, Brooks N, Yuan W, McNair C, Chand SN, et al. Targeting the p300/CBP axis in lethal prostate cancer. Cancer Discov. 2021;11:1118–37. 33431496 10.1158/2159-8290.CD-20-0751 PMC8102310

[37] Wang Y, Yao M, Li C, Yang K, Qin X, Xu L, et al. Targeting ST8SIA6-AS1 counteracts KRAS(G12C) inhibitor resistance through abolishing the reciprocal activation of PLK1/c-Myc signaling. Exp Hematol Oncol. 2023;12:105. 38104151 10.1186/s40164-023-00466-3 PMC10724920

[38] Bishop TR, subramanian C, Bilotta EM, Garnar-Wortzel L, Ramos AR, Zhang Y, et al. Acetyl-CoA biosynthesis drives resistance to histone acetyltransferase inhibition. Nat Chem Biol. 2023;19:1215–22. 37127754 10.1038/s41589-023-01320-7 PMC10538425

[39] Bauer K, Hauswirth A, Gleixner KV, Greiner G, Thaler J, Bettelheim P, et al. BRD4 degraders may effectively counteract therapeutic resistance of leukemic stem cells in AML and ALL. Am J Hematol. 2024;99:1721–31. 38822666 10.1002/ajh.27385

[40] Liu S, Zhou J, Ye X, Chen D, Chen W, Lin Y, et al. A novel lncRNA SNHG29 regulates EP300- related histone acetylation modification and inhibits FLT3-ITD AML development. Leukemia. 2023;37:1421–34. 37157016 10.1038/s41375-023-01923-y

[41] Zhang Y, Yuan L. Fms-like tyrosine kinase 3-internal tandem duplications epigenetically activates checkpoint kinase 1 in acute myeloid leukemia cells. Sci Rep. 2021;11:13236. 34168220 10.1038/s41598-021-92566-5 PMC8225911

[42] Cho MH, Park JH, Choi HJ, Park MK, Won HY, Park YJ, et al. DOT1L cooperates with the c-Myc-p300 complex to epigenetically derepress CDH1 transcription factors in breast cancer progression. Nat Commun. 2015;6:7821. 26199140 10.1038/ncomms8821 PMC4525167

[43] Ullius A, Lüscher-Firzlaff J, Costa IG, Walsemann G, Forst AH, Gusmao EG, et al. The interaction of MYC with the trithorax protein ASH2L promotes gene transcription by regulating H3K27 modification. Nucleic Acids Res. 2014;42:6901–20. 24782528 10.1093/nar/gku312 PMC4066752

[44] Liang J, Zubovitz J, Petrocelli T, Kotchetkov R, Connor MK, Han K, et al. PKB/Akt phosphorylates p27, impairs nuclear import of p27 and opposes p27-mediated G1 arrest. Nat Med. 2002;8:1153–60. 12244302 10.1038/nm761

[45] Weller S, Toennießen A, Schaefer B, Beigl T, Muenchow A, Böpple K, et al. The BCL-2 inhibitor ABT-199/venetoclax synergizes with proteasome inhibition via transactivation of the MCL-1 antagonist NOXA. Cell Death Discov. 2022;8:215. 35443750 10.1038/s41420-022-01009-1 PMC9021261

[46] Yao MY, Wang YF, Zhao Y, Ling LJ, He Y, Wen J, et al. BCL-2 inhibitor synergizes with PI3Kδ inhibitor and overcomes FLT3 inhibitor resistance in acute myeloid leukaemia. Am J Cancer Res. 2022;12:3829–42. 36119822 PMC9442011

[47] Hou P, Wu C, Wang Y, Qi R, Bhavanasi D, Zuo Z, et al. A genome-wide CRISPR screen identifies genes critical for resistance to FLT3 inhibitor AC220. Cancer Res. 2017;77:4402–13. 28625976 10.1158/0008-5472.CAN-16-1627 PMC5559306

[48] Attar N, Kurdistani SK. Exploitation of EP300 and CREBBP lysine acetyltransferases by cancer. Cold Spring Harb Perspect Med. 2017;7:a026534. 27881443 10.1101/cshperspect.a026534 PMC5334244

[49] Iyer NG, Ozdag H, Caldas C. p300/CBP and cancer. Oncogene. 2004;23:4225–31. 15156177 10.1038/sj.onc.1207118

[50] Giotopoulos G, Chan WI, Horton SJ, Ruau D, Gallipoli P, Fowler A, et al. The epigenetic regulators CBP and p300 facilitate leukemogenesis and represent therapeutic targets in acute myeloid leukemia. Oncogene. 2016;35:279–89. 25893291 10.1038/onc.2015.92 PMC4729186

[51] Raisner R, Kharbanda S, Jin L, Jeng E, Chan E, Merchant M, et al. Enhancer activity requires CBP/P300 bromodomain-dependent histone H3K27 acetylation. Cell Rep. 2018;24:1722–9. 30110629 10.1016/j.celrep.2018.07.041

[52] Bedford DC, Kasper LH, Fukuyama T, Brindle PK. Target gene context influences the transcriptional requirement for the KAT3 family of CBP and p300 histone acetyltransferases. Epigenetics. 2010;5:9–15. 20110770 10.4161/epi.5.1.10449 PMC2829352

[53] Daver N, Cortes J, Ravandi F, Patel KP, Burger JA, Konopleva M, et al. Secondary mutations as mediators of resistance to targeted therapy in leukemia. Blood. 2015;125:3236–45. 25795921 10.1182/blood-2014-10-605808 PMC4440880

[54] He Y, Sun L, Xu Y, Fu L, Li Y, Bao X, et al. Combined inhibition of PI3Kdelta and FLT3 signaling exerts synergistic antitumor activity and overcomes acquired drug resistance in FLT3-activated acute myeloid leukemia. Cancer Lett. 2018;420:49–59. 29409989 10.1016/j.canlet.2018.01.071

[55] Kesarwani M, Huber E, Azam M. Overcoming AC220 resistance of FLT3-ITD by SAR302503. Blood Cancer J. 2013;3:e138. 23995047 10.1038/bcj.2013.40 PMC3763391

[56] Ding L, Ley TJ, Larson DE, Miller CA, Koboldt DC, Welch JS, et al. Clonal evolution in relapsed acute myeloid leukaemia revealed by whole-genome sequencing. Nature. 2012;481:506–10. 22237025 10.1038/nature10738 PMC3267864

[57] Hnisz D, Abraham BJ, Lee TI, Lau A, Saint-André V, Sigova AA, et al. Super-enhancers in the control of cell identity and disease. Cell. 2013;155:934–47. 24119843 10.1016/j.cell.2013.09.053 PMC3841062

