# Supplementary material for: Concurrent inhibition of p300/CBP and FLT3 enhances cytotoxicity and overcomes resistance in acute myeloid leukemia
Source: Acta Pharmacol Sin. 2025 Jan 30;46(5):1390–403. doi: 10.1038/s41401-025-01479-w (PMC12032420; doi:10.1038/s41401-025-01479-w)
Supplement: Supplementary file 1 — Supplementary Figure legends [file 41401_2025_1479_MOESM1_ESM.docx]

**Supplementary Figures**

**Fig. S1** **In vitro sensitivity of AML cell lines to p300/CBP inhibitors**.

**(a)** Analysis of p300 paralog CREB-binding protein (*CREBBP*) gene expression profile in different tumor samples and paired normal tissue samples in the GEPIA database. Relative data were obtained from the TCGA dataset. TPM, transcripts per million. **(b)** Analysis of *CREBBP* mRNA expression levels in AML and normal tissue samples in the GEPIA database. Relative data were obtained from the TCGA and GTEx datasets. **(c)** AML cell lines were treated with increasing concentrations of CCS1477, A485 and quizartinib for 72 h. IC_50_ values were calculated using Prism 5 software. **(d)** Cell cycle analyses of MV-4-11, RS4;11 and HEL cell lines after 24 h treatment of 250 nM A485. **(e)** Quantification data in (**d**) were shown graphically and summarized as means ± SD from three independent experiments. ***P*< 0.01.

**Fig. S2 EP300/CBP inhibitors and quizartinib exhibit synergistic effects on FLT3 signaling and H3K27Ac regulation.**

**(a)** The relative protein levels of c-Myc, p300, FLT3 and H3K27Ac in Fig. 2g were quantified and shown. **(b)** The relative protein levels of c-Myc, H3K27Ac, P-STAT5 and P-ERK in Fig. 3a were quantified and shown. **(c)** The relative protein levels of c-Myc and p300 in Fig. 3b were quantified and shown. **(d)** The relative protein levels of c-Myc in Fig. 3c were quantified and shown. **(e)** The relative protein levels of c-Myc, P-FLT3, FLT3 and H3K27Ac in Fig. 3e were quantified and shown. The data are presented as means ± SD, n = 3. Statistical significance was assessed using two-tailed unpaired Student’s t test. **P* < 0.05, ***P* < 0.01, and ****P* < 0.001, ns indicates not significant.

**Fig. S3 The combination of** **p300/CBP inhibitors and** **quizartinib exhibits synergistic antileukemic activity.**

Cell cycle phase distributions of cells treated with indicated concentrations of quizartinib, A485 (**a**), or CCS1477 (**b**) either alone or in combination for 24 h were determined by flow cytometry. The data are shown with the representative results from three biological replicates. **(c)** The relative protein levels of P-CDK2, CDK2, p27 and Cyclin D1 in Fig. 3i were quantified and shown. **(d)** The online ChIP-seq data of MOLM-13 cells after exposure to 3 μM A485 for 2 h (from GEO database: GSE211051) were analyzed. KEGG analysis showing the enrichment of relative signaling pathways between A485 treatment and DMSO control group. **(e)** GO analysis showing the enrichment of biological process between A485 treatment and DMSO control group. **(f)** The relative protein levels of c-Myc, P-STAT5, P-ERK and H3K27Ac in Fig. 3n were quantified and shown. The data are presented as means ± SD, n = 3. Statistical significance was assessed using two-tailed unpaired Student’s t test. **P* < 0.05, ***P* < 0.01, and ****P* < 0.001.

**Fig. S4 Differential expression profiles of genes associated with p300/CBP in MV-4-11 and MV-4-11/quizartinib AML cells**.

**(a)** Heatmap cluster analysis showing the differentially expressed genes (DEGs) in MV-4-11 cells and MV-4-11/quizartinib cells treated with DMSO or 20 nM quizartinib for 6 h. **(b)** Union of statistically significant upregulated and downregulated DEGs in MV-4-11 and MV-4-11/quizartinib cells treated with DMSO or quizartinib. Each treatment involved three independent biological replicates. **(c)** KEGG pathway analysis of the genes associated with transcription regulator in MV-4-11/quizartinib cells compared to parental cells. **(d)** The relative levels of indicated proteins in Fig. 4f were quantified and shown. **(e)** The relative protein levels of c-Myc, P-FLT3 and H3K27Ac in Fig. 4g were quantified and shown. **(f)** The relative protein levels of c-Myc, p300, FLT3 and H3K27Ac in Fig. 4h were quantified and shown. The data are presented as means ± SD, n = 3. Statistical significance was assessed using two-tailed unpaired Student’s t test. **P* < 0.05, ***P* < 0.01, and ****P* < 0.001, ns indicates not significant.

**Fig. S5 In vitro activity of MV-4-11/quizartinib cells to quizartinib**.

**(a**) MV-4-11 and MV-4-11/quizartinib cells were treated with increasing concentrations of quizartinib or tubulin inhibitor paclitaxel for 72 h. MTT assays were performed to determine cell viability. The data are presented as means ± SD from three independent experiments. **(b)** Resistance factors for quizartinib, A485, CCS1477 and paclitaxel in MV-4-11/quizartinib cells compared to MV-4-11 cells were calculated according to their IC_50_ values. **(c)**, **(d)** Cell cycle profiles of MV-4-11 and MV-4-11/quizartinib cells after 24 h treatment of quizartinib. **(e)** The relative protein levels of c-Myc, P-FLT3 and H3K27Ac in Fig. 5b were quantified and shown. **(f)** The relative protein levels of P-CDK2, CDK2, p27 and Cyclin D1 in Fig. 5g were quantified and shown. **(g)** The relative protein levels of c-Myc, P-FLT3, FLT3 and H3K27Ac in Fig. 5h were quantified and shown. **(h)** Nude mice were engrafted with MV-4-11/quizartinib cells and treated with vehicle, A485 (100 mg/kg), quizartinib (1 mg/kg), or their combination for 14 days. Tumor photos were shown. **(i)** The relative protein levels of c-Myc, P-STAT5, P-ERK and H3K27Ac in Fig. 5l were quantified and shown. The data are graphically and summarized as means ± SD from three independent experiments. **P* < 0.05, ***P* < 0.01, ****P* < 0.001, ns indicates not significant.

**Fig. S6 The combination of p300/CBP inhibitors and quizartinib exhibits synergistic effect in primary FLT3-ITD^+^ AML samples**.

**(a), (b)** The relative protein levels of c-Myc, P-STAT5, P-ERK and H3K27Ac in Fig. 6d were quantified and shown. The data are graphically and summarized as means ± SD from three independent experiments. **P* < 0.05, ***P* < 0.01, ****P* < 0.001.
