# Supplementary figures and images for: Concurrent inhibition of p300/CBP and FLT3 enhances cytotoxicity and overcomes resistance in acute myeloid leukemia
Source: Acta Pharmacol Sin. 2025 Jan 30;46(5):1390–403. doi: 10.1038/s41401-025-01479-w (PMC12032420; doi:10.1038/s41401-025-01479-w)

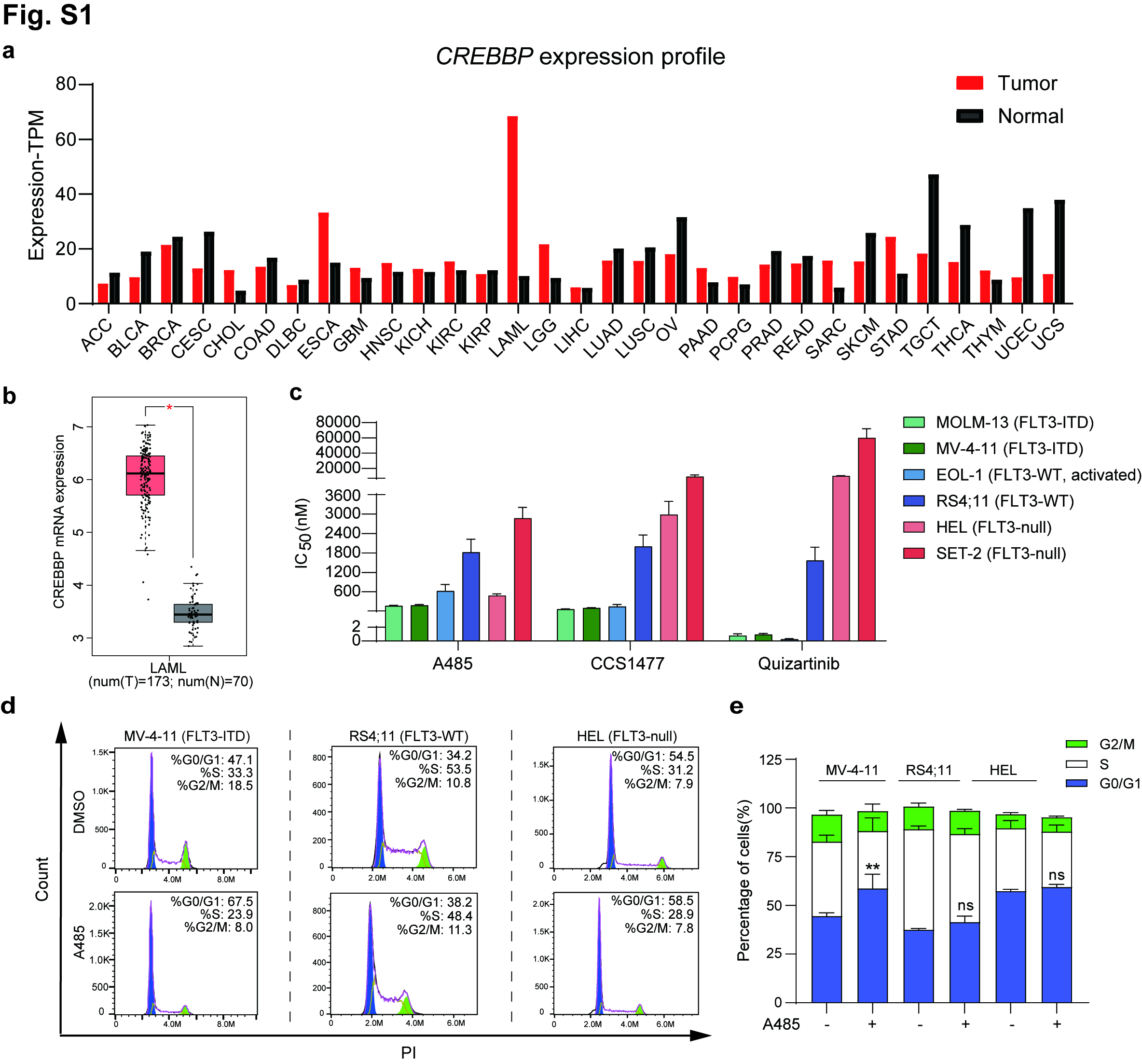

Supplement: Supplementary file 2 — Supplementary Figure S1 [file 41401_2025_1479_MOESM2_ESM.tif]

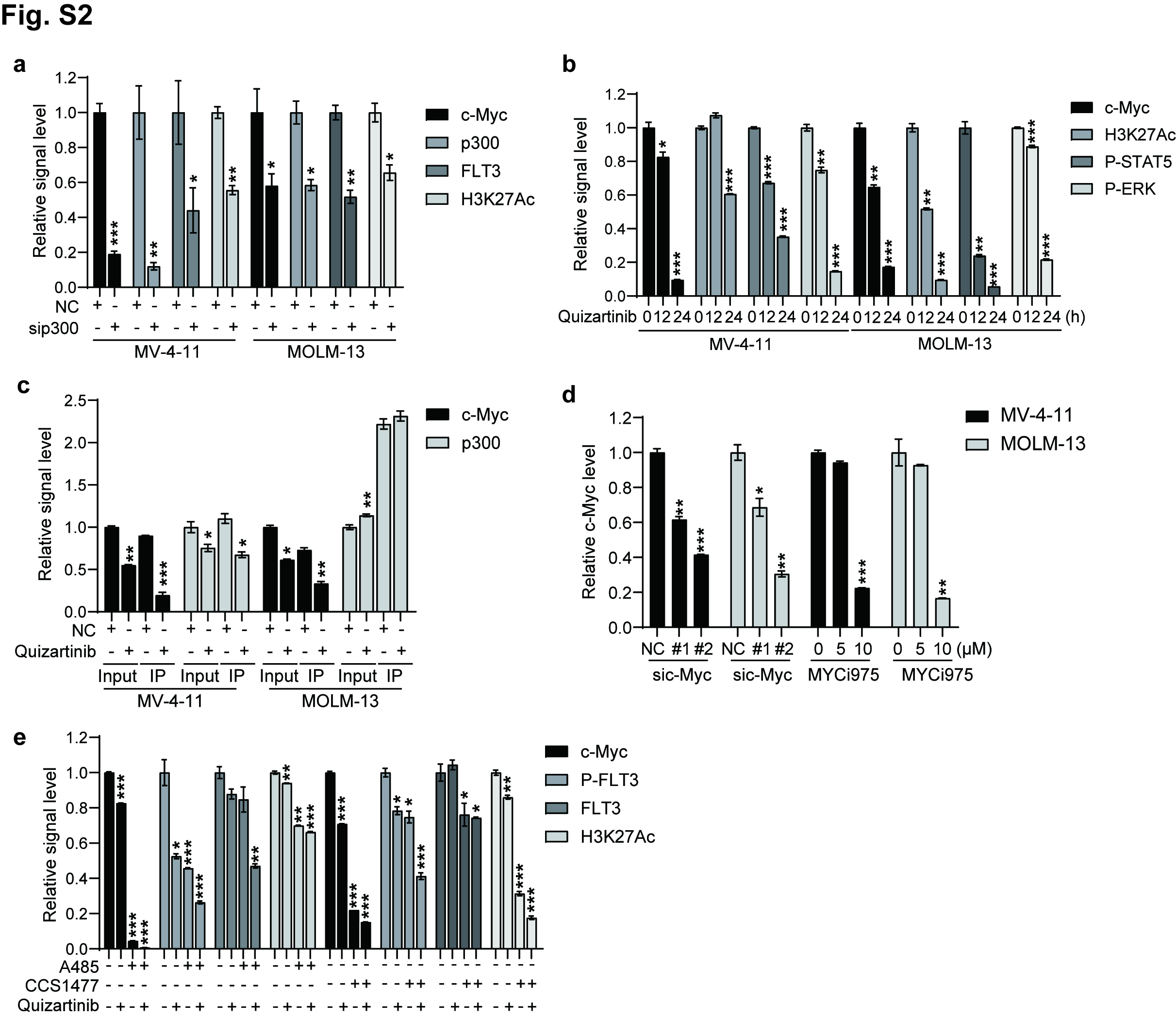

Supplement: Supplementary file 3 — Supplementary Figure S2 [file 41401_2025_1479_MOESM3_ESM.tif]

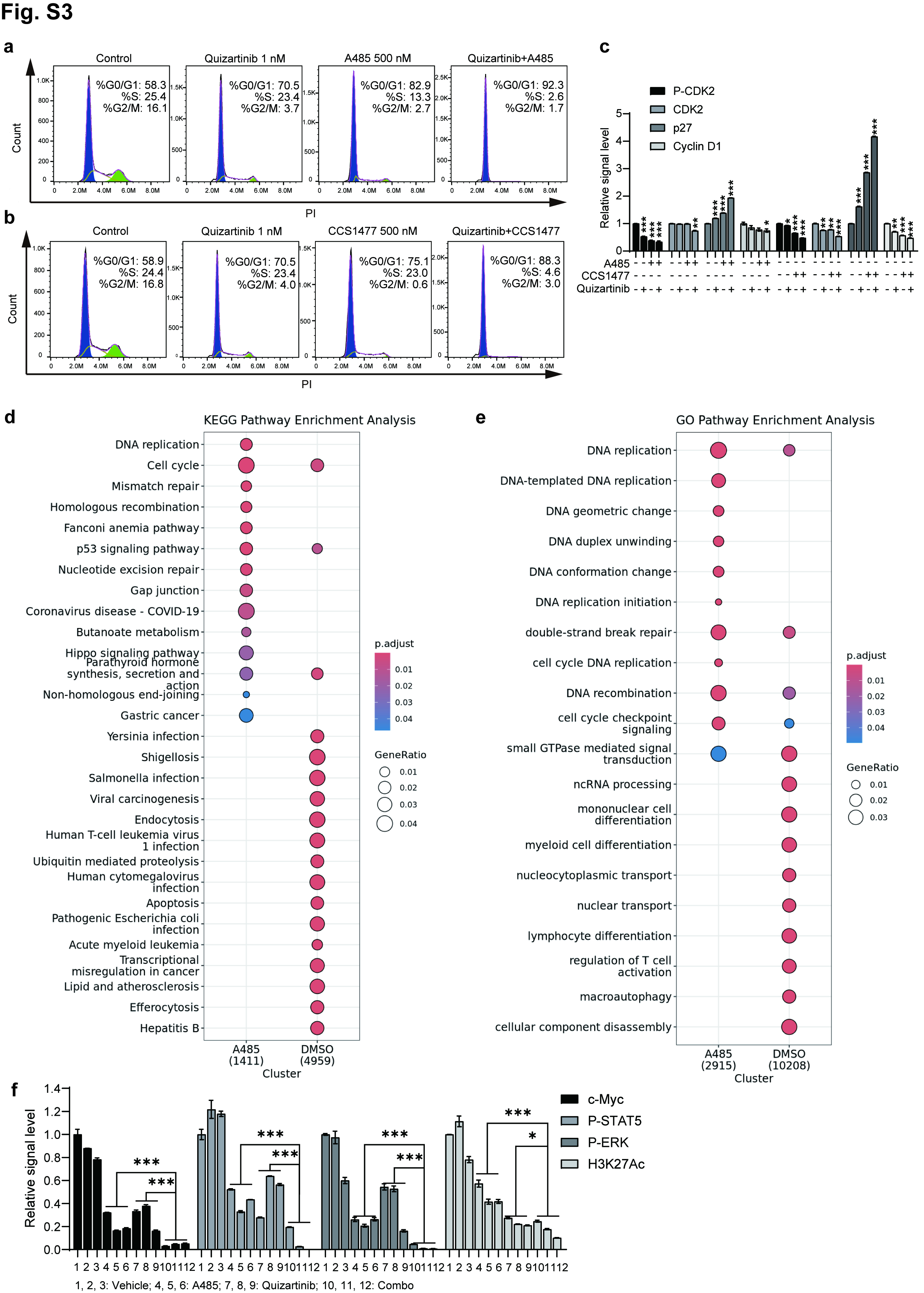

Supplement: Supplementary file 4 — Supplementary Figure S3 [file 41401_2025_1479_MOESM4_ESM.tif]

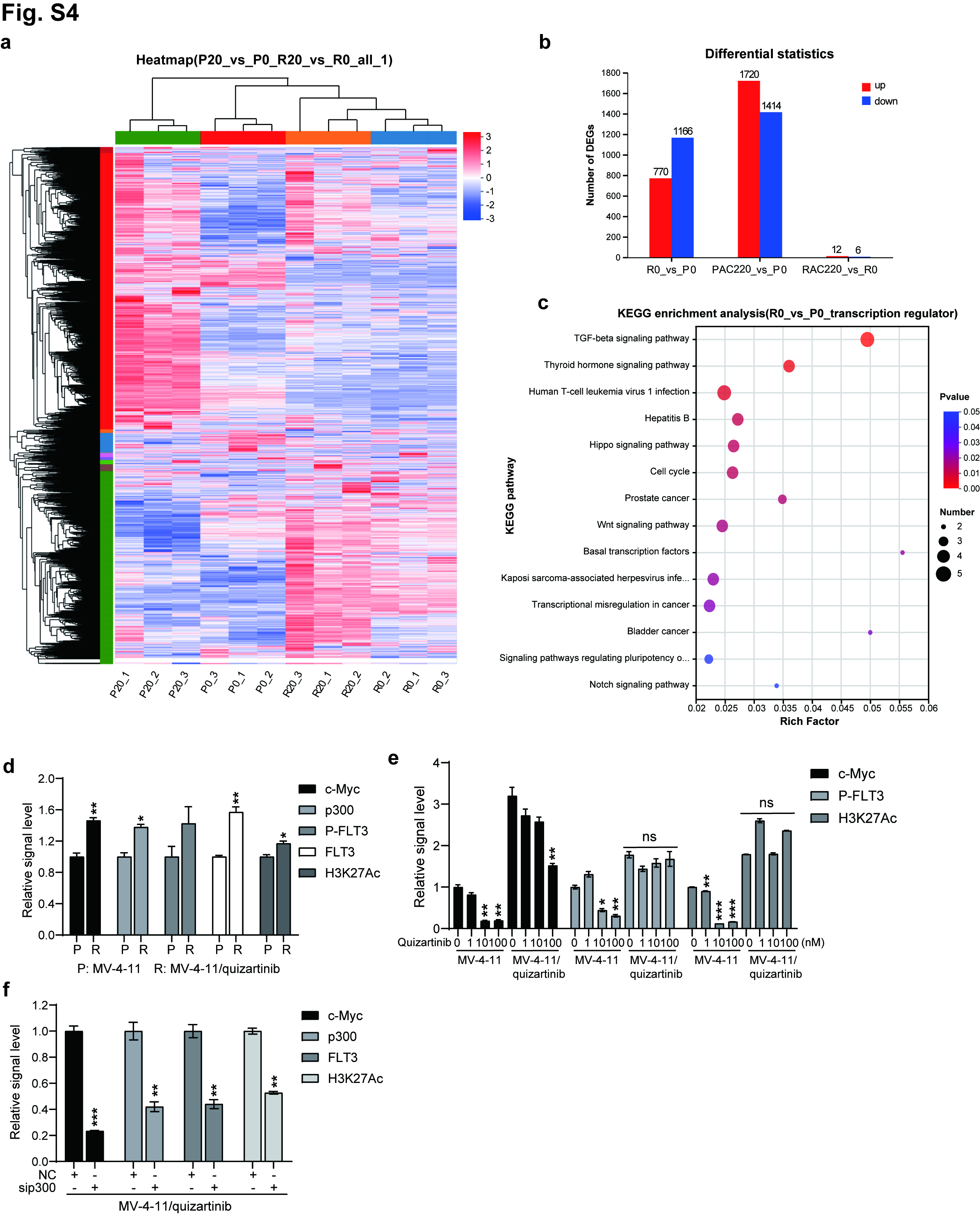

Supplement: Supplementary file 5 — Supplementary Figure S4 [file 41401_2025_1479_MOESM5_ESM.tif]

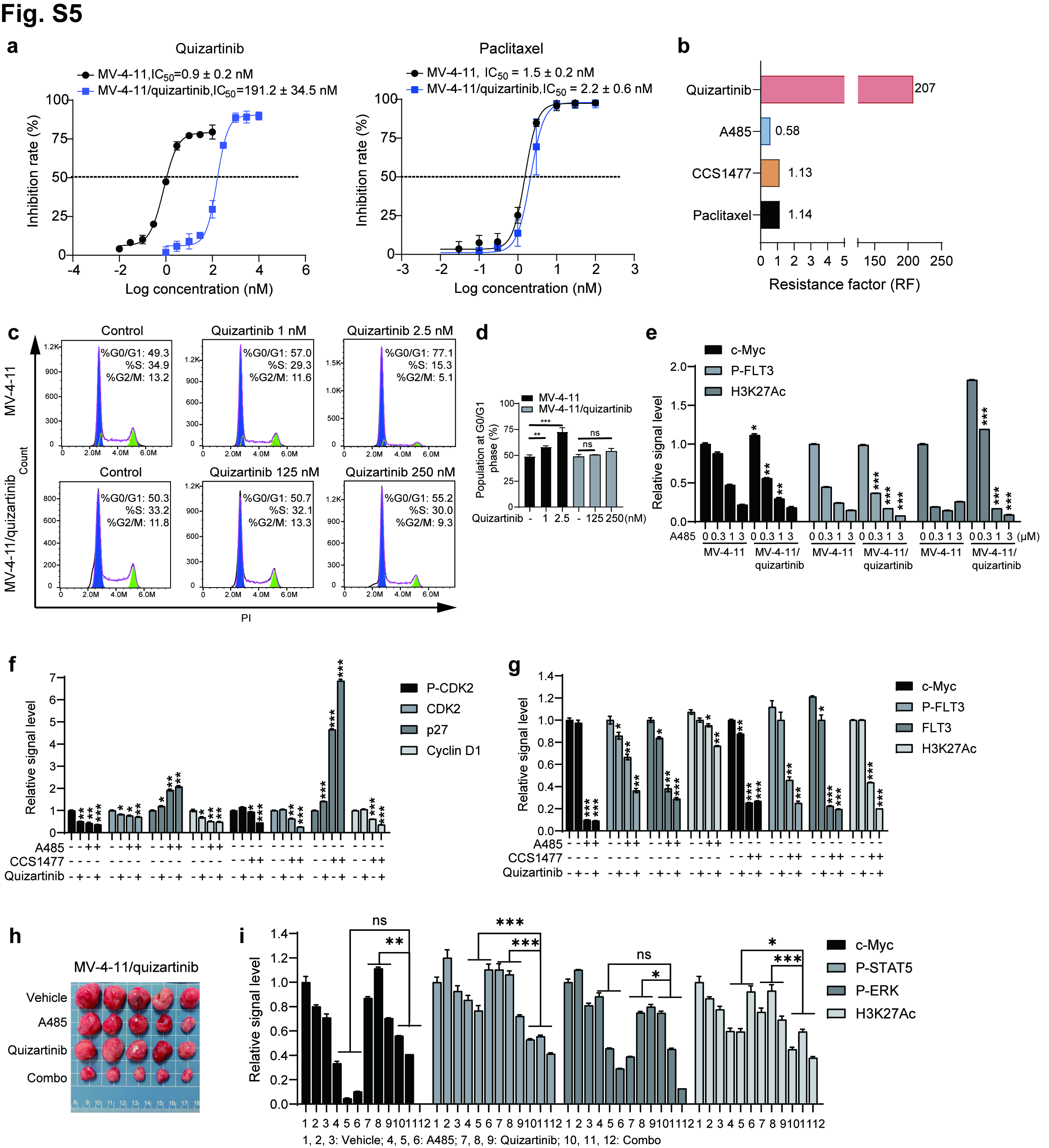

Supplement: Supplementary file 6 — Supplementary Figure S5 [file 41401_2025_1479_MOESM6_ESM.tif]

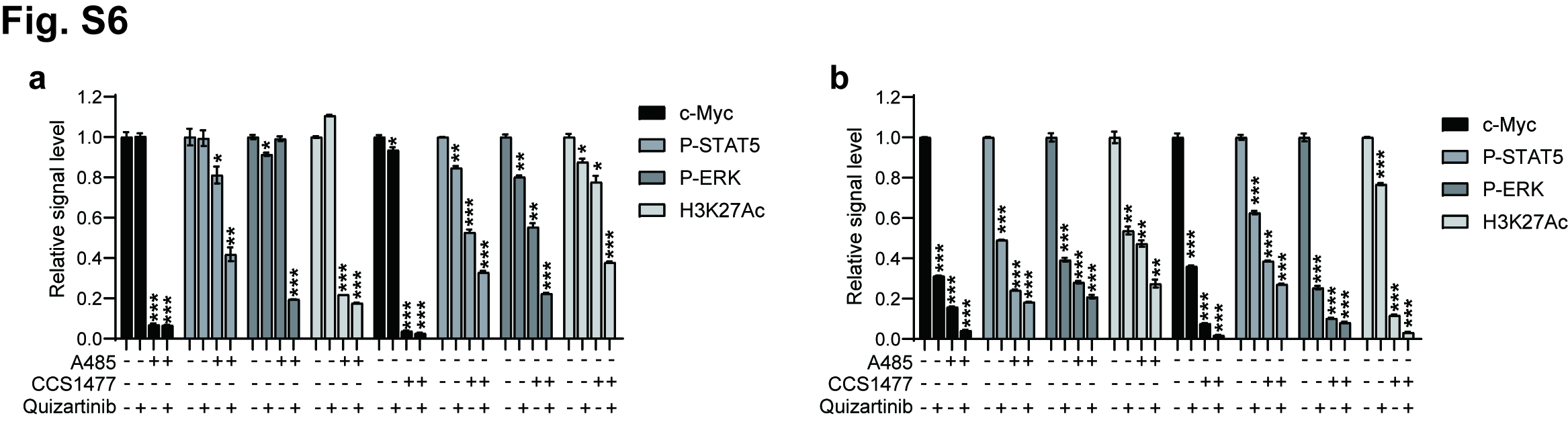

Supplement: Supplementary file 7 — Supplementary Figure S6 [file 41401_2025_1479_MOESM7_ESM.tif]
