# Supplementary material for: Concurrent inhibition of p300/CBP and FLT3 enhances cytotoxicity and overcomes resistance in acute myeloid leukemia
Source: Acta Pharmacol Sin. 2025 Jan 30;46(5):1390–403. doi: 10.1038/s41401-025-01479-w (PMC12032420; doi:10.1038/s41401-025-01479-w)
Supplement: Supplementary file 8 — Supplementary Tables [file 41401_2025_1479_MOESM8_ESM.docx]

**Supplementary Tables**

**Table S1** Primers used in RT-qPCR.

| Gene | Forward primer | Reverse primer |
| --- | --- | --- |
| *EP300* | GCTTCAGACAAGTCTTGGCAT | ACTACCAGATCGCAGCAATTC |
| *KAT2B* | GAAAAACCCTAACCCCTCACC | CCTTGTGGACACAGGTAAAGAGA |
| *CEBPB* | ACCAACCGCACATGCAGAT | GCAGAGGGAGAAGCAGAGAGTTT |
| *FLT3* | CGAGGAGGGCAACTACTTTGAGATG | TGACTGGGATGCTTTGAAGAGGAAC |
| *MYC* | GGCTCCTGGCAAAAGGTCA | CTGCGTAGTTGTGCTGATGT |
| *GAPDH* | GACATCAAGAAGGGGTGAA | TGTCATACCAGGAAATGAGC |

**Table S2** Primers used in ChIP-qPCR.

| Gene | Forward primer | Reverse primer |
| --- | --- | --- |
| *FLT3* pro1 | TGCTGCTTTCAACACGGGTGTA | TCTACCATGGTGCAGCCACTAT |
| *FLT3* pro2 | AAGTAGCTACGACTACAGTGC | ATCATTTGAGGTCAGGAGTTGG |

**Table S3** Clinical and biological features of primary AML patient samples.

| AML patients | Age | Gender | FLT3 status | ITD/WT |
| --- | --- | --- | --- | --- |
| # 1 | 63 | Male | ITD | 0.81 |
| # 2 | 32 | Male | ITD | 1.03 |
| # 3 | 66 | Female | ITD | 2.75 |
| # 4 | 57 | Male | ITD | 0.002 |

ITD/WT: FLT3-ITD allelic ratio

**Table S4** Cell proliferation of MV-4-11 and MV-4-11/quizartinib cells.

| Targets | Inhibitors | IC_50_ (nM; mean ± SD) | | RF |
| --- | --- | --- | --- | --- |
|  |  | MV-4-11 | MV-4-11/quizartinib |  |
| FLT3 | quizartinib | 0.92 ± 0.18 | 191.23 ± 34.54 | 207.86 |
| p300/CBP | A485 | 175.27 ± 34.16 | 101.14 ± 7.94 | 0.58 |
| p300/CBP | CCS1477 | 193.50 ± 10.47 | 218.40 ± 76.65 | 1.13 |
| Tubulin | Paclitaxel | 1.83 ± 0.53 | 2.09 ± 0.82 | 1.14 |

*RF: resistance factor; SD: standard deviation
